# The NAD kinase OsNADK1 affects the intracellular redox balance and enhances the tolerance of rice to drought

**DOI:** 10.1186/s12870-019-2234-8

**Published:** 2020-01-07

**Authors:** Xiang Wang, Bin-Bin Li, Tian-Tian Ma, Liang-Yu Sun, Li Tai, Chun-Hong Hu, Wen-Ting Liu, Wen-Qiang Li, Kun-Ming Chen

**Affiliations:** 1 State Key Laboratory of Crop Stress Biology in Arid Area/College of Life Sciences, Northwest A&F University, Yangling, 712100 Shaanxi China; 2 CAS Key Laboratory of Plant Germplasm Enhancement and Specialty Agriculture, Wuhan Botanical Garden, Chinese Academy of Sciences, Wuhan, 430074 Hubei China; 3 College of Life Science and Agriculture, Zhoukou Normal University, Zhoukou, 466001 Henan China

**Keywords:** NAD kinase OsNADK1, Intracellular redox balance, OsDREB1B, Proline, Drought tolerance, Rice (*Oryza sativa*)

## Abstract

**Background:**

NAD kinases (NADKs) are the only known enzymes that directly phosphorylate NAD(H) to generate NADP(H) in different subcellular compartments. They participate in multiple life activities, such as modulating the NADP/NAD ratio, maintaining the intracellular redox balance and responding to environmental stresses. However, the functions of individual NADK in plants are still under investigation. Here, a rice NADK, namely, OsNADK1, was identified, and its functions in plant growth regulation and stress tolerance were analysed by employing a series of transgenic plant lines.

**Results:**

OsNADK1 is a cytosol-localized NADK in rice. It was expressed in all rice tissues examined, and its transcriptional expression could be stimulated by a number of environmental stress treatments. Compared with wild-type (WT) rice, the mutant plant *osnadk1* in which *OsNADK1* was knocked out was a dwarf at the heading stage and had decreased NADP(H)/NAD(H), ascorbic acid (ASA)/dehydroascorbate (DHA) and reduced glutathione (GSH)/oxidized glutathione (GSSG) ratios, which led to increased oxidation states in the rice cells and sensitivity to drought. Moreover, certain stress-related genes showed differential expression patterns in *osnadk1* under both normal growth and drought-stress conditions compared with WT. Among these genes, *OsDREB1B* and several WRKY family transcription factors, e.g., *OsWRKY21* and *OsWRKY42*, showed correlated co-expression patterns with *OsNADK1* in *osnadk1* and the plants overexpressing or underexpressing *OsNADK1*, implying roles for these transcription factors in OsNADK1-mediated processes. In addition, overexpression of *OsNADK1* enhanced the drought tolerance of rice plants, whereas loss of function of the gene reduced the tolerance. Furthermore, the proline content was dramatically increased in the leaves of the *OsNADK1*-overexpressing lines under drought conditions.

**Conclusions:**

Altogether, the results suggest that an OsNADK1-mediated intracellular redox balance is involved in the tolerance of rice plants to drought.

## Background

NAD kinases (NADKs) are the only known enzymes that generate NADP(H) by phosphorylating NAD(H) [1–3] and, therefore, play vital roles in maintaining the balance between NAD(H) and NADP(H) in NADP(H)-based metabolic cellular pathways [4, 5]. NADKs have been identified in almost every living organism, including Archaea, Eubacteria and Eukaryotes, although not in the intracellular parasite *Chlamydia trachomatis* [2]. However, the number of NADK isoforms varies depending on the species. Archaea and Eubacteria usually have only one NADK, but most Eukaryotes have multiple NADKs [2], and plants have at least three [6]. These NADKs exist in different subcellular locations, including the cytosol, chloroplast, mitochondria and/or peroxisome [4, 7–12], showing their diverse functions.

The functions of NADKs in various organisms have been widely studied, including those from *Escherichia coli* [13], *Saccharomyces cerevisiae* [7, 14], *Arabidopsis thaliana* [15–17], rice [18], and wheat [12]. For *Mycobacterium tuberculosis* and *Salmonella enterica*, mutation of specific sites in their single NADK gene is lethal [2, 19]. Similarly, mutating all three NADK genes encoding Utr1,Yef1, and Pos5 or the two genes encoding Utr1 and Pos5 together in *S. cerevisiae* is also lethal [20, 21], which again indicates that NADK activity is necessary for cell survival. In *Arabidopsis*, a single mutation in one of the three NADKs is not lethal, but it greatly affects plant growth [15, 16, 22]. In rice, loss of function of *OsNADK3* leads to dwarfism and sterility [18]. These results support the fundamental roles of NADKs in the development of most living organisms.

In recent years, the protection afforded by NADKs against oxidative-stress damage has received much attention because NADPH is a central component of the intracellular antioxidative defence system [15, 16, 23, 24]. In *S. cerevisiae*, the activities of three NADKs, namely, Utr1, Yef1, and Pos5, contribute to NADPH supplementation and thereby help protect yeast cells from oxidative damage [25, 26]. This antioxidative function has also been found in animal NADKs [9, 27]. In plants, Berrin and colleagues found that deletion of *AtNADK1,* the encoded NADK of which is localized in the cytosol, enhanced the sensitivity of *Arabidopsis* to gamma irradiation and paraquat-induced oxidative stress [10, 22]. Chai et al. and Sun et al. showed that a gene knockout mutant of AtNADK2, which mainly exists in the chloroplast, shows inhibited plant growth and substantially increased sensitivity to drought [15, 28]. A knockout mutant of AtNADK3 localized to peroxisomes is also more sensitive to oxidative stress than the wild-type plant [10, 16, 29]. More recently, we found that transcriptional expression of NADK genes in wheat, rice, and maize could be differentially stimulated by a number of environmental stress and hormonal treatments, implying their certain roles in the multi-stress responses of plants [6, 12, 30].

The three intracellular plant redox couples, namely, NADPH/NADP, reduced glutathione (GSH)/oxidized glutathione (GSSG) and ascorbic acid (ASA)/dehydroascorbate (DHA), appear to play the most important roles in environmental stress responses [31, 32]. In *Pseudomonas fluorescens*, NADK substantially enhances NADPH biosynthesis and thereby diminishes oxidative damage induced by environmental stress [33]. In rat pancreatic β-cells, Gray and colleagues found that NADK regulates the size of the NADPH pool and the NADPH/NADP^+^ ratio, thereby influencing the intracellular redox balance and oxidative defence system [27]. In *Arabidopsis*, a loss of function mutation in *AtNADK2* was found to cause an increment in NAD(H) levels but a decrement in NADP(H) levels in chloroplasts [15, 34–36]. A decrement in GSH/GSSG values was also observed in *atnadk3*, an Arabidopsis *AtNADK3* knockout mutant [16]. Additionally, overexpression of *AtNADK2* in rice enhanced the GSH/GSSG ratio and NADP(H) pool of cells, leading to high tolerance of the plants to oxidative damage [36]. More recently, we reported that NADKs may play important roles in maintaining reactive oxygen species (ROS) homeostasis in different subcellular compartments of plant cells by regulating NADPH production and intercellular redox status [30]. Calmodulin may act as a fundamental regulator of NADK-meditated NAD signalling in plant development and the response to stress [37].

Although many studies have addressed the roles of plant NADKs, their functional characteristics and mechanisms in response to abiotic stresses have not been completely elucidated. As reported previously, there are at least four NADKs in the three major cereal crops, namely, rice, maize and wheat, and these NADKs are differentially compartmentalized in cells [6, 12]. In the present study, a rice NADK gene, *OsNADK1*, was cloned, and its functions in maintaining the intracellular redox balance and stress tolerance were explored. The obtained results suggest that OsNADK1 is related to the intracellular redox balance of rice and may play a role in drought stress tolerance of the plant.

## Results

### Expression profiles of *OsNADK1* in different tissues and under various external conditions

**Fig. 1:**
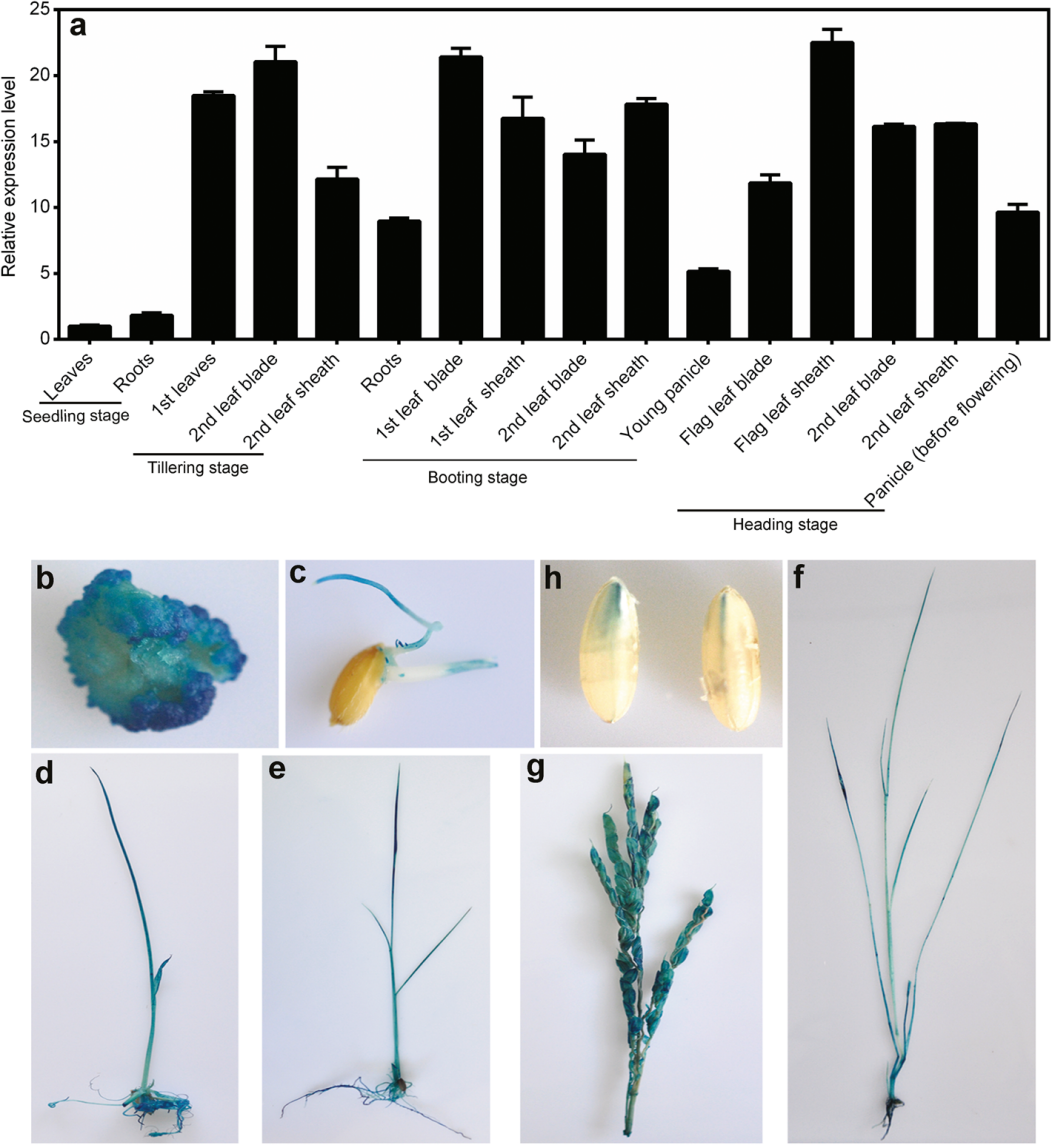
Expression pattern of *OsNADK1* in different tissues at various developmental stages. **a** Analysis of *OsNADK1* expression levels in various tissues by qRT-PCR. *OsActin1* and *OsUBQ* were used as the internal controls. **b**-**h** Histochemical localization of the *OsNADK1* promoter: GUS expression pattern in transgenic rice plants: (**b**) callus, (**c**) radicle and germ, (**d**) one-week-old seedlings, (**e**) two-week-old seedlings, (**f**) one-month-old seedlings, (**g**) young panicles, and (**h**) maturing seeds

*OsNADK1* expression profiles in different tissues and under various stress conditions were examined by quantitative real-time RT-PCR (qRT-PCR). As shown in Fig. 1a, *OsNADK1* transcripts were detected in almost all examined tissues, i.e., roots, leaves, leaf blades, leaf sheaths and panicles, although a higher transcript level was observed in the leaf blade and leaf sheath during the different developmental stages.

We then employed a β-glucuronidase (GUS) reporter assay to further characterize the expression pattern of *OsNADK1* in various rice tissues during development. A 2000-bp nucleotide sequence upstream of the *OsNADK1* coding sequence that contains the *OsNADK1* promoter region was cloned into the pCAMBIA1301 vector (instead of the 35S promoter controlling the GUS gene), and histochemical staining of GUS activity in the *OsNADK1* promoter: GUS transgenic plants was detected. Strong GUS staining was observed in each of the examined tissues, including the calli, radicle and germ, young panicles, maturing seeds, and 1-week-, 2-week-, and 1-month-old seedlings, showing the activity of the *OsNADK1* promoter in all the analysed tissues (Fig. 1b-h).

**Fig. 2:**
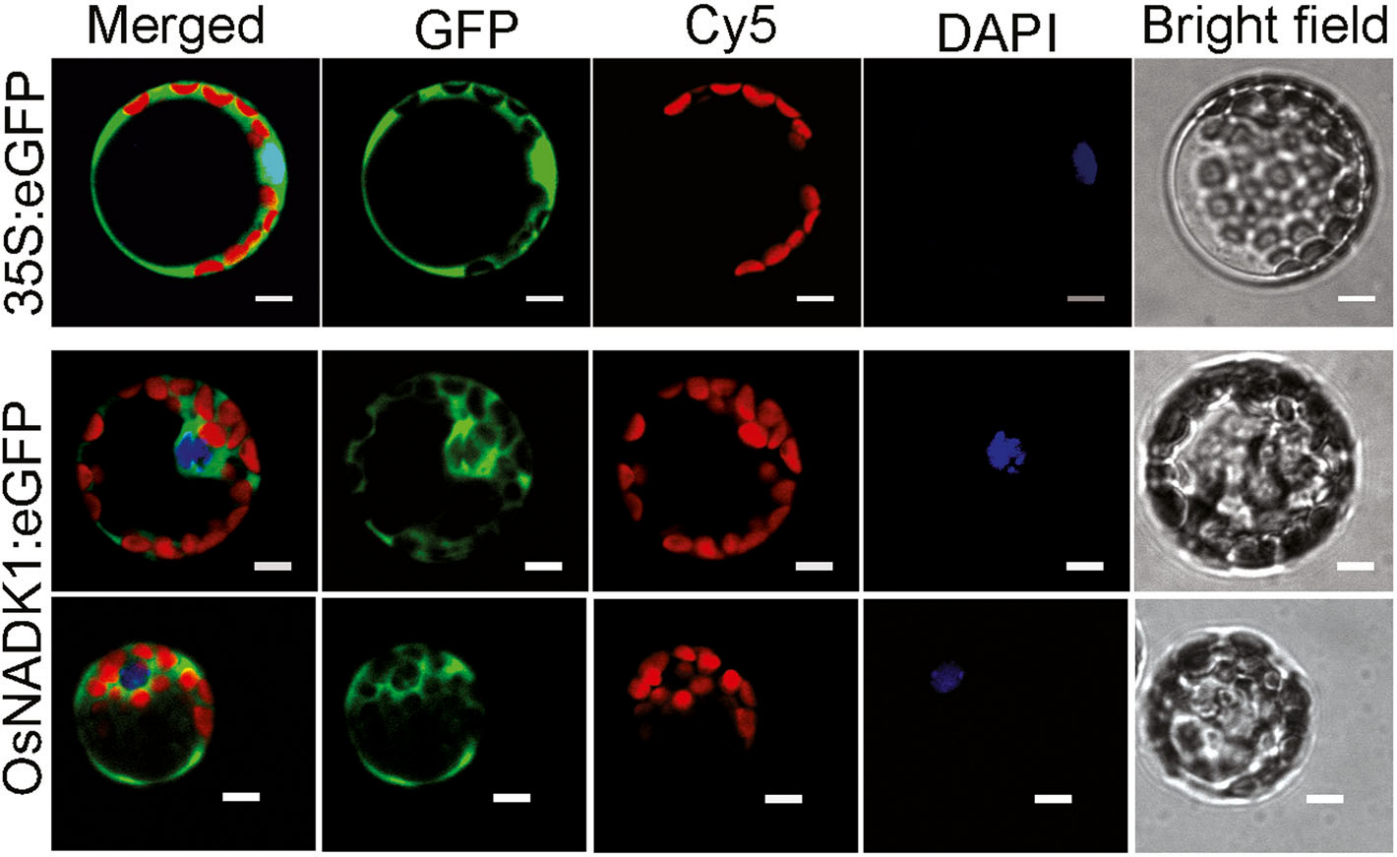
Subcellular localization of OsNADK1 in rice protoplasts. In each panel, the extreme right shows the bright field; then, DAPI represents the nuclear location by staining with a nucleus (N)-specific dye, Cy5 is the chloroplast spontaneous fluorescence, the left shows the GFP field, and the extreme left shows an overlay of the three images to the right. Bars = 5 μm

Based on a PlantCare analysis, many cis-elements that are responsive to various abiotic stresses and hormones, including drought (MBS), heat (HSE), anaerobic conditions (ARE), methyl jasmonic acid (MeJA) (CGTCA-motif), abscisic acid (ABA) (ABRE), ethylene (ERE), gibberellin (p-box) and auxin (TGA-element), were identified in the promoter region of *OsNADK1* (Additional file 1: Figure S1). Analysis by qRT-PCR showed that the *OsNADK1* transcripts were greatly stimulated 3.9-, 92-, 22-fold by cold (4 °C), oxidative stress (30 μM methyl viologen (MV) exposure) and dehydration (20% (w/v) PEG-6000) after a 12-h treatment of shoots, as well as 3.4-, 4.0-, 4.1-fold by cold, heat (40 °C), and salt (exposure to 200 mM NaCl) (Additional file 2: Figure S2) in roots, respectively. In addition, OsNADK1 is located in the cytoplasm where the green fluorescence of OsNADK1-green fluorescent protein (GFP) was found in rice protoplasts (Fig. 2).

### Characterization of *osnadk1*, an *OsNADK1*-knockout mutant

**Fig. 3:**
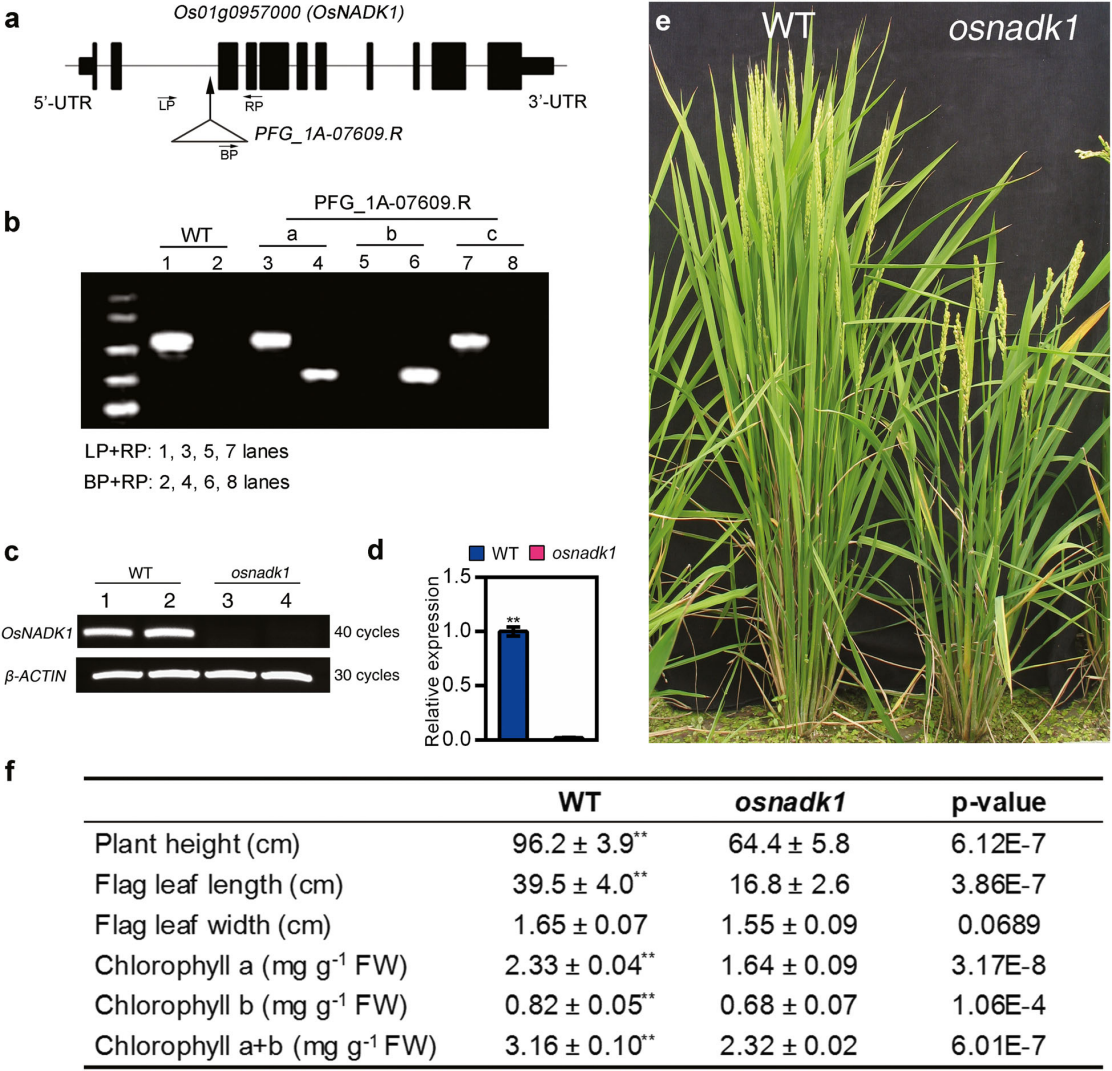
Identification of the *osnadk1* mutant and morphology of WT (cv. Dongjin) and *osnadk1* plants. **a** The gene structure of *OsNADK1* (not to scale). Solid boxes and lines indicate exons and introns, respectively. The arrow indicates the T-DNA insertion position, and the triangle represents the T-DNA insertion. “LP”, “BP” and “RP” are the positions of primers for identification. **b** Screening of the *osnadk1* mutant by PCR. Lanes 1, 3, 5, 7 show amplicons generated using the left and right primers (LP and RP, respectively) described in Methods; lanes 2, 4, 6, 8 show amplicons generated using the right primer and T-DNA-border primer (BP) described in Methods. a-c, Plants grown from *PFG_1A-07609.R* seeds. **c** The *OsNADK1* transcript levels in WT and *osnadk1* plants detected by semi-quantitative RT-PCR. *β-ACTIN* was used as a control. Lanes 1 and 2, WT; lanes 3 and 4, *osnadk1*. **d** The expression levels of *OsNADK1* in the mutant and WT tested by qRT-PCR. **e** and **f** Gross morphology and agronomic characteristics of WT and *osnadk1* plants at the early filling stage of growth. Values represent the mean ± SD of eight replicates. The letters denote significant differences (*p* ≤ 0.05) according to t-test analysis

To further understand the functions of OsNADK1, we characterized the *OsNADK1*-knockout mutant *osnadk1* derived from the T-DNA insertion rice mutant *PFG_1A-07609.R*. The mutant and its wild-type (WT) parent (cv. Dongjin) were purchased from Pohang University of Science and Technology, Pohang, Korea. *OsNADK1* contains 11 exons and 10 introns, and the T-DNA insertion site in *OsNADK1* is located within the second intron (Fig. 3a). Southern blotting analysis showed that a single insertion was present in the mutated gene (Additional file 3: Figure S3). The genotype of the mutated rice gene was further characterized by PCR using the forward (LP) and reverse (RP) primers and a T-DNA-border primer (BP). The results showed that the plant named “b” was a homozygote since only one band was obtained using BP plus RP primers, while no bands were detected using LP plus RP primers (Fig. 3b). Both semi-quantitative PCR and qRT-PCR analyses showed that the mutant *osnadk1* did not express *OsNADK1* (Fig. 3c, d), indicating that it contained a null mutation of *OsNADK1*. Compared with the WT plants, *osnadk1* had a reduced height, smaller flag-leaf dimensions and lower chlorophyll contents (Fig. 3e, f) at the early filling stage of plant growth.

### Drought tolerance of *osnadk1* plants

**Fig. 4:**
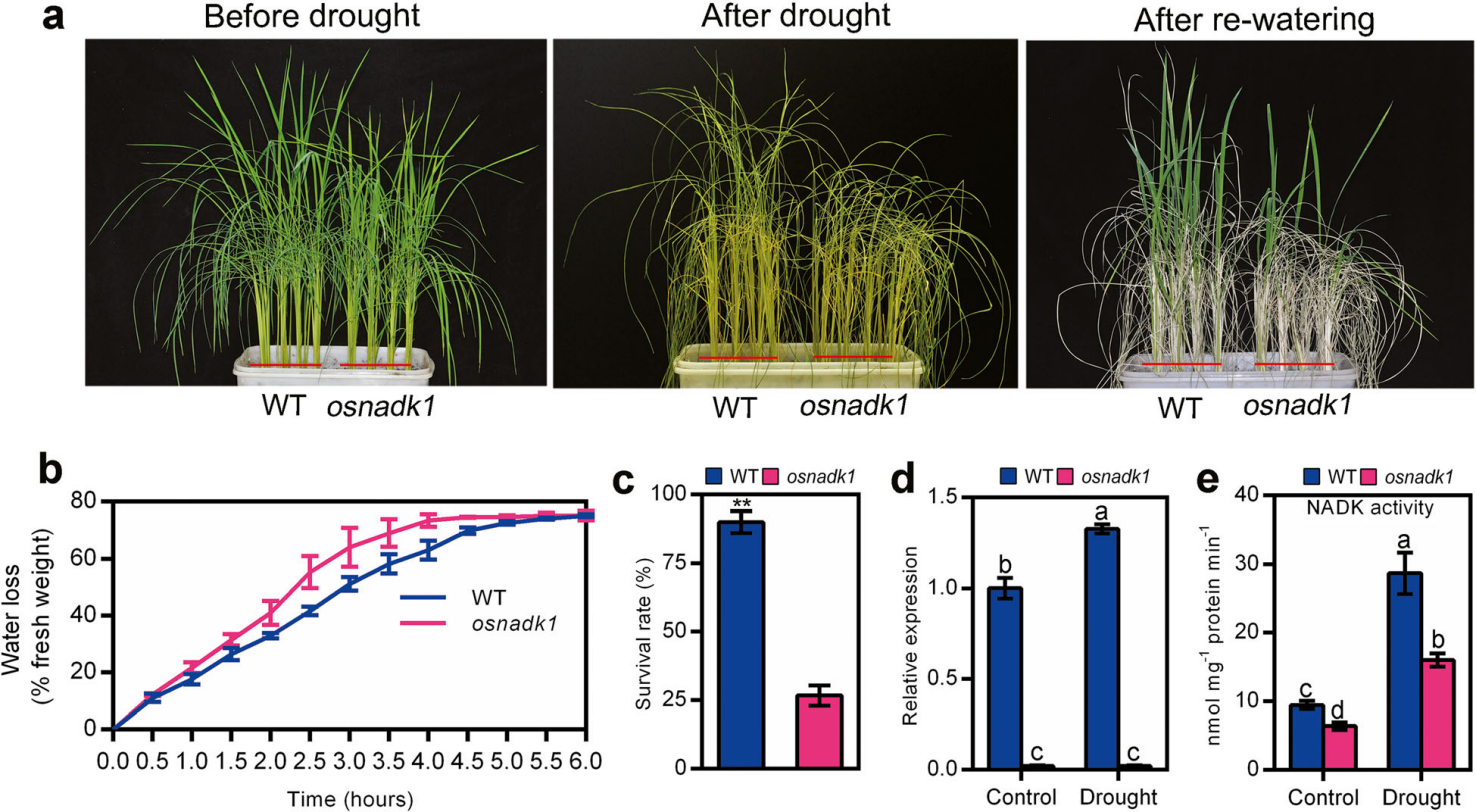
Drought tolerance of wild-type (WT, cv. Dongjin) and *osnadk1* mutant plants. **a** Phenotypes of WT and *osnadk1* rice plants under drought conditions, including before drought treatment (left image), without watering for 10 days (middle image), and re-watering after 1 week (right image). Four-week-old plants were used for the drought treatments. **b** Water loss percentage in detached leaves of WT and *osnadk1* mutant plants. Values represent the mean ± SD of 3 replicates (9 leaves from one pot per replicate), and three experiments were performed with similar results. **c** Survival rate of WT and *osnadk1* mutant plants under drought conditions. **d** Comparison of the relative expression levels of *OsNADK1* in the WT and *osnadk1* mutant under control and drought conditions. **e** Total NADK activity in leaf samples from the WT and *osnadk1* mutant. Samples were collected from leaves without watering for 10 days and control samples. ** Represents significant differences by the t-test, and different letters above the bars represent significant differences by the Tukey method (*P* ≤ 0.05)

To gain further insights in the function of OsNADK1, the drought tolerance of *osnadk1* was carefully examined. Four-week-old plants were used for the drought treatment experiments. As seen in Fig. 4, the null mutant, *osnadk1*, was sensitive to drought-stress, and after 10 days of drought treatment, most of the mutant plants had died, with only a few surviving after rewatering (Fig. 4a). The survival rate of *osnadk1* was merely 29.6% of that of WT (Fig. 4c). Based on a general consideration that plants with higher water retention ability can survive better under drought conditions, we then checked the water loss rate of WT and *osnadk1* mutant. As expected, the mutant plants possessed a faster water loss rate compared with the WT plants (Fig. 4b).

We also tested the expression level of *OsNADK1* and the total NADK activity in the mutant and WT under drought conditions. As seen, the expression level of *OsNADK1* was increased 1.35-fold in WT under drought conditions, while the *osnadk1* mutant showed no *OsNADK1* expression under both normal growth and drought conditions (Fig. 4d). The total NADK activity was increased considerably both in WT and *osnadk1* under drought conditions, but it was markedly lower in the *osnadk1* mutant compared with WT (Fig. 4e). The total NADK activity of the mutant was only 67.9 and 55.8% of that of WT under normal growth and drought conditions, respectively.

### Comparison of the intracellular redox balance in *osnadk1* and WT

**Fig. 5:**
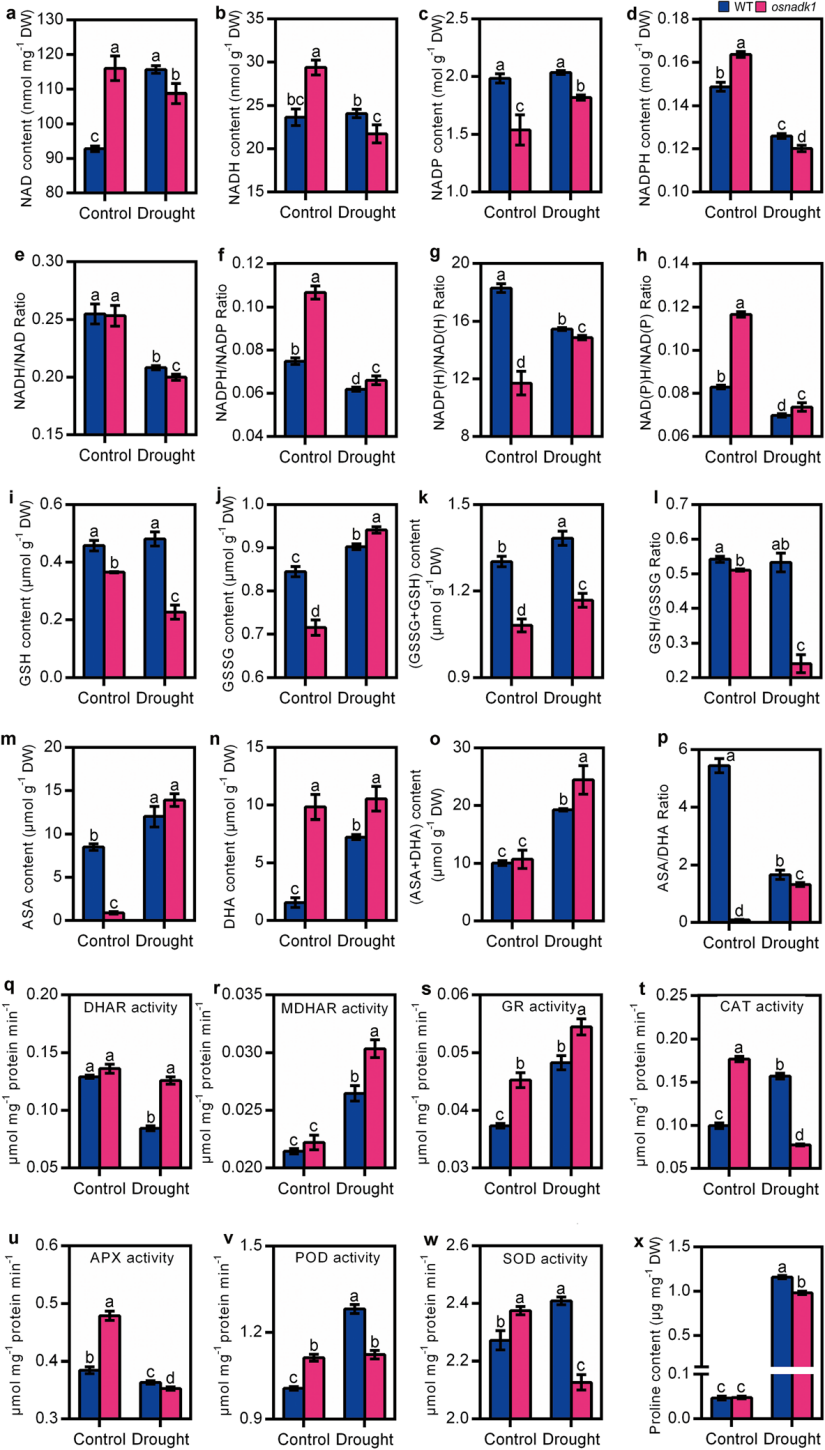
Intracellular redox status, antioxidant enzyme activity and proline content of the wild-type (WT, cv. Dongjin) and *osnadk1* mutant under normal growth and drought stress conditions. Four-week-old plants were used for the drought treatment, and after 10 days of treatment, the leaves were used for the analysis. **a**-**d** The contents of NAD, NADH, NADP and NADPH. **i**-**k** The contents of GSH, GSSG and total glutathione (GSSG+GSH). **m**-**o** The contents of ASA, DHA and total ascorbic acid (ASA + DHA). **e**-**h**, **j**, **p** The ratios of NADH/NAD, NADPH/NADP, NADP(H)/NAD(H), NAD(P)H/NAD(P), GSH/GSSG, and ASA/DHA, respectively. **q**-**w** The enzyme activities of DHAR, MDHAR, GR, CAT, APX, POD and SOD, respectively. **x** The content of proline. All data are means ± SD and are representative of similar results from three independent experiments. Different letters above the bars represent significant differences by the Tukey method (*P* ≤ 0.05)

To gain further insights into the OsNADK1 functions in rice, the states of the major intracellular redox couples, namely, NADPH/NADP, GSH/GSSG and ASA/DHA, were characterized in *osnadk1* and WT. Under normal growth conditions, the levels of NAD and NADH in *osnadk1* were both greater than WT, while the NADH/NAD ratio was not different between the mutant and WT (Fig. 5a, b, e). However, the amount of NADP was lower in *osnadk1* than in WT, while the amount of NADPH was greater in the mutant (Fig. 5c, d), leading to a higher NADPH/NADP ratio of *osnadk1* (Fig. 5f). Notably, the ratio of NADP(H)/NAD(H) was also lower in the *osnadk1* mutant (Fig. 5g). The NADP(H)/NAD(H) ratio in the mutant was merely 64.0% of that in WT. However, the NAD(P)H/NAD(P) ratio in *osnadk1* was higher and showed a nearly 50% increase in the mutant compared with WT (Fig. 5h). In addition, excluding the content of NADP, which was increased by drought in the *osnadk1* mutant, the contents of the other three indexes, namely, NAD, NADH and NADPH, were all decreased after drought compared with normal conditions (Fig. 5a-d). Similar results were also found for the ratios of NADH/NAD, NADPH/NAD, and NAD(P)H/NAD(P). The three ratios were all decreased after drought in both the *osnadk1* mutant and WT (Fig. 5e, f, h). An exception was observed in the ratio of NADP(H)/NAD(H), which was increased by drought in *osnadk1* compared with WT (Fig. 5g).

The states of the other two redox couples in *osnadk1* and WT were similar to NADPH/NADP under normal growth conditions, with lower concentrations of GSH, GSSG and ASA and a higher concentration of DHA in the mutant than WT (Fig. 5i, j, m, n). The total amounts of GSSG and GSH were reduced in the mutant, but the total concentrations of ASA and DHA showed no apparent difference between WT and *osnadk1* under control conditions (Fig. 5k, o). However, the ratios of the reductants to oxidants for the ASA/DHA and GSH/GSSG redox couples were markedly lower in *osnadk1* than WT (Fig. 5l, p). After drought treatment, excluding the content of GSSG, which was slightly higher in *osnadk1* than in WT, the three other items including the contents of GSH and total glutathione (GSH + GSSG) and the ratio of GSH/GSSG were highly reduced in the mutant compared with WT. The content of ASA showed no apparent difference in WT and *osnadk1* under drought, although it increased in both the mutant and WT in response to the stress treatment (Fig. 5m). However, the contents of DHA and total ascorbic acid (ASA + DHA) were higher in the mutant than WT after drought (Fig. 5). In contrast, the ASA/DHA ratio was markedly lower in the *osnadk1* mutant than WT under both normal growth and drought stress conditions (Fig. 5p). All these results indicated that the intracellular redox balance was markedly altered when *OsNADK1* was knocked out in rice under both normal growth and drought stress conditions.

In addition, the activities of catalase (CAT), peroxidase (POD) and superoxide dismutase (SOD), as well as enzymes including glutathione reductase (GR), ascorbate peroxidase (APX), dehydroascorbate reductase (DHAR) and monodehydroascorbate reductase (MDHAR) that function in the Halliwell-Asada cycle (ASA-GSH cycle) together with the three redox couples in plant cells [38], were increased in *osnadk1* compared with WT, especially under normal growth conditions (Fig. 5q-w). However, although the activities of MDHAR, GR, CAT, POD and SOD were particularly increased in WT by drought, only the activities of MDHAR, DHAR and GR were much higher in the *osnadk1* mutant than in WT, implying that the antioxidative ability was harmed in the mutant (Fig. 5q-w). We also checked the content of proline in WT and *osnadk1* under normal growth and drought conditions. Although the content of proline was higher in WT after drought, it showed no apparent difference between the mutant and WT under normal growth conditions (Fig. 5x).

### Global gene expression in *osnadk1* and WT under normal growth and drought conditions

**Fig. 6:**
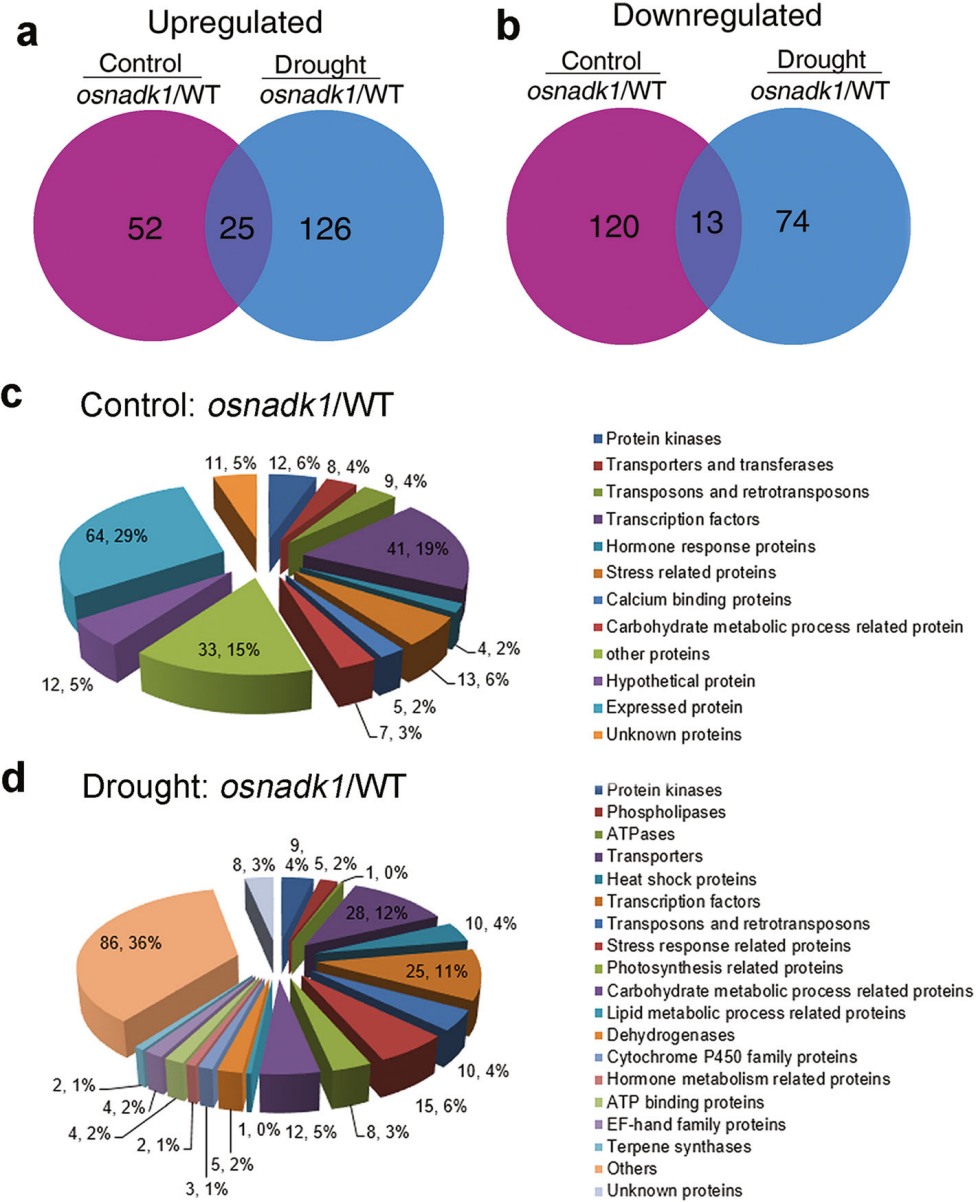
Gene-chip Venn diagrams and functional classification pie charts by function for genes impacted by ablation of *OsNADK1* and drought treatment. **a** and **b** Differences in gene expression between *osnadk1* and WT (cv. Dongjin) plants under normal growth and drought conditions. Number of upregulated (**a**) or downregulated (**b**) genes in *osnadk1* under normal (purple) or drought (blue) conditions and under both experimental conditions (crossing of purple and blue), respectively. **c** and **d** The functional classification of genes that were differentially expressed in *osnadk1* and WT under normal conditions (**c**) and drought conditions (**d**), respectively. The gene number and percentage for each functional category are shown. Only genes for which expression changed by two or more fold (*p* ≤ 0.05) were used to draw the Venn diagrams and pie charts. The values are averages of three independent replicates. Control: soil moisture, 47.3%; drought: soil moisture, 8.5%

**Fig. 7:**
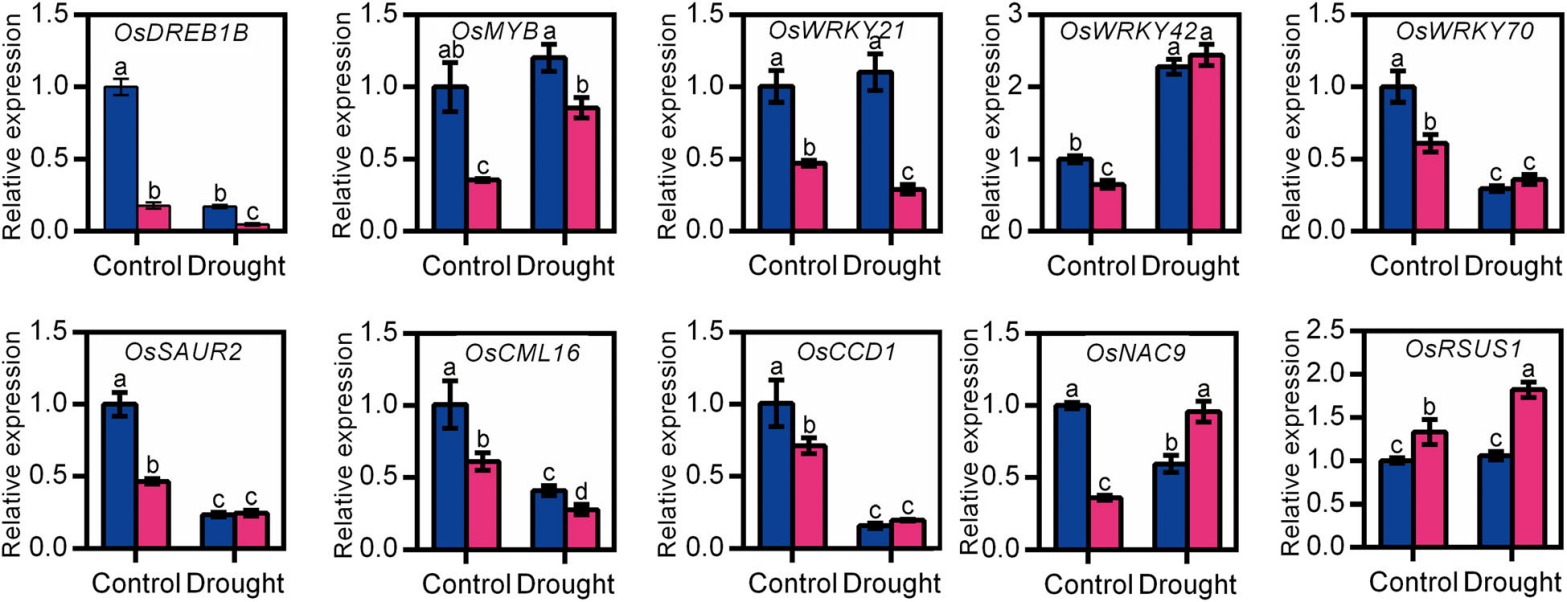
Relative expression levels of several genes that may be related to *OsNADK1* expression in the wild-type (WT, cv. Dongjin) and *osnadk1* mutant under normal growth and drought stress conditions. Four-week-old plants were used for the drought treatment. The gene symbols and primer sequences used for qRT-PCR are listed in Additional file 8: Table S3. Control, watering normally; drought, without watering for 10 days. Data are means ±SD from three independent biological replicates. Bars annotated with different letters represent values that were significantly different by the Tukey method (*p* ≤ 0.05)

Due to the sensitivity of the *osnadk1* mutant to drought, the global gene-expression profiles of *osnadk1* under normal growth and drought conditions were investigated using Affymetrix GeneChip rice-genome arrays. A substantial number of genes (210 in total) were differentially expressed in *osnadk1* compared with WT (cv. Dongjin) even when the plants are grown under normal (non-stressed) growth conditions (Additional file 6: Table S1). The expression levels of those genes were changed more than two-fold (*p* ≤ 0.05), with 77 genes upregulated and 133 downregulated (Additional file 6:Table S1; Fig. 6a, b). For the plants under drought stressed, a total of 238 genes were differentially regulated more than two-fold in *osnadk1,* with 151 upregulated and 87 downregulated compared with WT (Additional file 7: Table S2; Fig. 6a, b). Based on the annotations in the Rice Annotation Project Database (http://rapdb.dna.affrc.go.jp/) and the MSU Rice Genome Annotation Project Database (http://rice.plantbiology.msu.edu/), the proteins encoded by these differentially expressed genes belonged to many different functional categories, including protein kinases, transporters, transferases, transposons and retrotransposons, transcription factors, ATPases, proteins responsive to stresses and those involved in many essential metabolic processes, e.g., lipid, carbohydrate, and hormone metabolism (Fig. 6c, d). Under normal growth conditions, 12 genes encoding protein kinases were differentially expressed in *osnadk1* compared with WT, in which the two DUF26 kinase-encoding genes were upregulated, whereas the others were downregulated (Additional file 6: Table S1). Among the 31 differentially regulated transcriptional factor-encoding genes, 28 genes were downregulated and only three upregulated in the mutant. However, nine genes encoding transposons or retrotransposons were all upregulated in the mutant. In addition, 10 genes that have been reported to participate in drought tolerance were selected for further analysis by qRT-PCR (Fig. 7). Among those genes, the three WRKY superfamily genes (*OsWRKY21*, *42* and *70*), a dehydration-responsive element-binding protein (OsDREB1B) gene, an auxin-responsive protein (OsSAUR2) gene and an MYB family transcription factor (OsMYB) gene were all appreciably downregulated in the mutant. Additionally, a gene encoding the calmodulin-related calcium sensor protein OsCML16, and a gene encoding an EF-hand Ca^2+^-binding protein named CCD1, were also severely downregulated in the mutant under normal growth conditions (Additional file 6: Table S1, Fig. 7).

Under drought conditions, a larger number of genes involved in more functional categories were differentially expressed in the mutant compared with WT. For example, several genes that were annotated as encoding cytochrome P450s and terpene synthases were differentially expressed in the mutant under drought conditions (Additional file 7: Table S2). Among the 28 genes encoding various transporters, 24 were upregulated in the mutant under drought. Excluding the two Ty3-gypsy subclass retrotransposon genes, which were downregulated in the mutant under drought, a total of eight other transposon and retrotransposon genes were markedly upregulated. In addition, a number of genes related to photosynthesis and carbohydrate, lipid and hormone metabolism were also differentially expressed in the mutant compared with WT under drought (Additional file 7: Table S2). Moreover, in contrast to normal growth conditions, the expression of *OsDREB1B*, *OsWRKY21, OsWRKY70, OsSAUR2*, *OsCML16* and *OsCCD1* was decreased in *osnadk1* by drought, whereas the expression of *OsMYB*, *OsWRKY42, OsNAC9* and *OsRSUS1* was increased in the mutant under this stress (Fig. 7).

### Generation of transgenic lines and their drought tolerance assessment

**Fig. 8:**
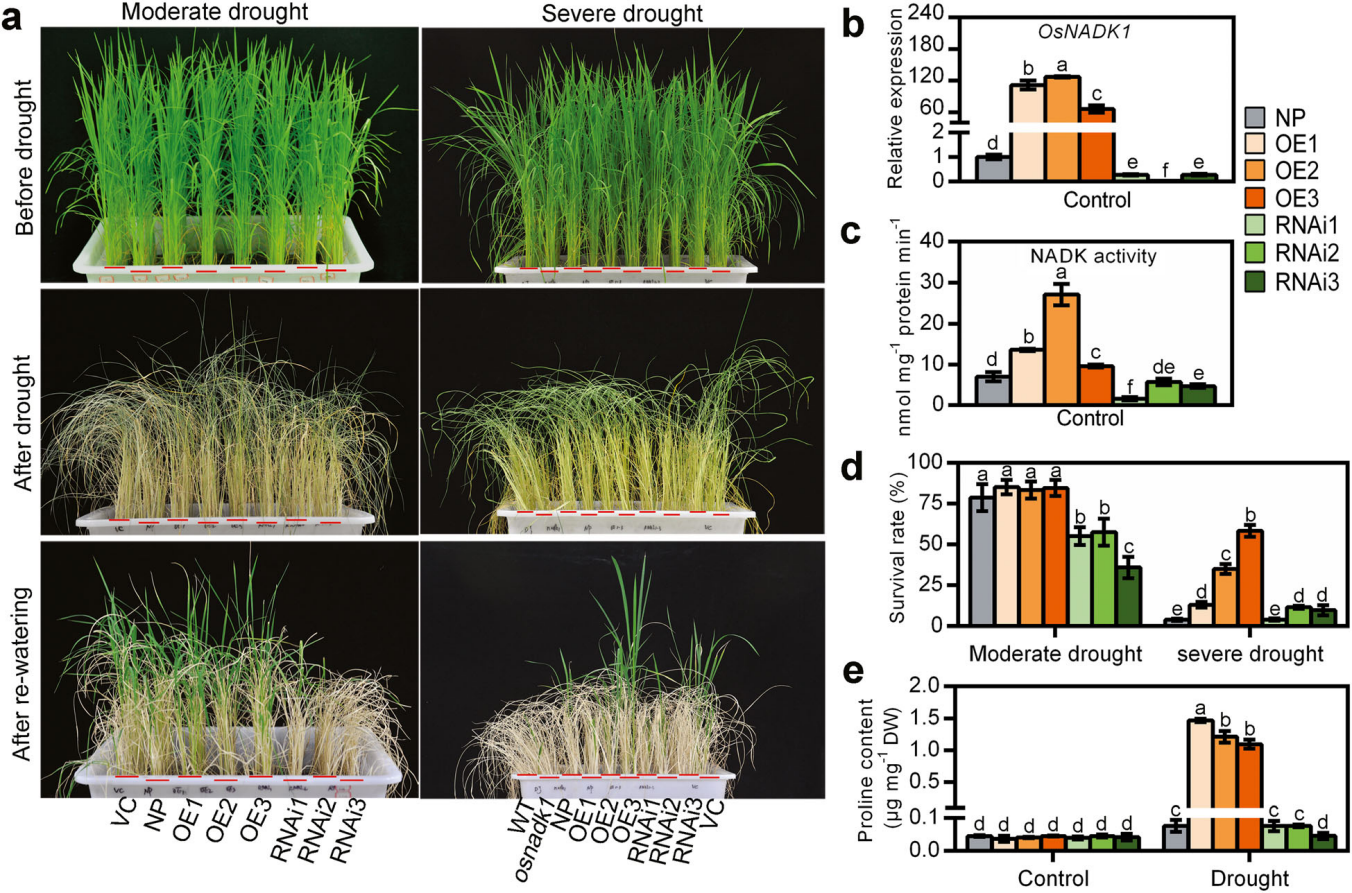
Drought tolerance of Nipponbare (NP), *OsNADK1*-overexpressing (OE) and RNA interference (RNAi) transgenic plants. Four-week-old plants were used for the drought treatment. **a** Phenotypes of the different types of plants before drought treatment (top images), without watering for 10 days (moderate drought) or 15 days (severe drought) (middle images), and with re-watering 1 week (moderate drought) or 2 weeks (severe drought) (bottom images). **b** The transcripts of *OsNADK1* in transgenic lines detected by qRT-PCR under normal conditions. The wild-type of cv. Nipponbare was used as the control. **c** Total NADK activity in leaf samples from the transgenic lines. Samples were collected from the leaves of the control. **d** Survival rates in the different lines. Data are means ±SD from two independent biological replicates. **e** Contents of proline (μg mg^− 1^ DW). WT, cv. Dongjin; NP: cv. Nipponbare; VC: vector control. Data are means±SD from three independent biological replicates. Bars annotated with different letters represent values that were significantly different by the Tukey method (*p* ≤ 0.05)

To further clarify the functions of *OsNADK1* in rice, the transgenic *OsNADK1*-overexpressing (OE) and RNA-interference (RNAi) rice lines were generated by an *Agrobacterium*-mediated transfection process using calli from the rice cv. Nipponbare. We obtained a total of 18 independent *OsNADK1*-overexpressing transgenic lines and 20 independent RNA-interference (RNAi) transgenic lines of rice. Three independently generated lines with various *OsNADK1* transcription levels for both the OE and RNAi plants (T2 generation) were selected for further study. The transcription levels of *OsNADK1* were increased by 110-, 130-, and 60-fold in the OE1, OE2, OE3 lines, and decreased by 0.2-, 0.03-, and 0.3-fold in the RNAi1, RNAi2, and RNAi3 lines, respectively (Fig. 8b). However, both types of transgenic plants exhibited no obvious differences in morphology under normal growth conditions compared with WT and the empty-vector transgenic control (VC) plants at the seedling stage (Fig. 8a). Consistent with the transcription level, the NADK activity was also increased in the OE lines but decreased in the RNAi lines (Fig. 8c). Then, four-week-old plants were used for drought treatment, and the transgenic plants showed differences in drought tolerance (Fig. 8a, d). As seen, after 10 days of drought (moderate drought), fewer than half of the RNAi plants were alive, showing their reduced tolerance to drought compared with the WT and OE lines. Following drought treatment for 15 days (severe drought), although the survival rates were not obviously different between the WT and RNAi plants, the OE plants exhibited a higher degree of drought tolerance. The survival rates of the OE2 and OE3 plants were much greater than the WT and RNAi lines. The amount of accumulated proline, which is thought to be associated with the drought tolerance of plants, was also measured in the OE and RNAi lines. Interestingly, although the amount of proline did not differ in the two types of transgenic plants compared with WT (cv. Nipponbare) under normal growth conditions, a higher level was detected in the OE plants under drought (Fig. 8e).

To further understand the roles of OsNADK1 in the intracellular redox balance, we then tested the redox related indexes in the transgenic plants (Additional file 4: Figure S4). The contents of NADP and ASA as well as the ratios of NADPH/NADP, NADP(H)/NAD(H), and NAD(P)H/NAD(P) in the RNAi lines were largely consistent with the *osnadk1* mutant (Additional file 4: Figure S4). By contrast, these components showed little change in the OE lines compared with WT, while the content of ASA and the ratio of ASA/DHA were greatly increased in the OE plants (Additional file 4: Figure S4). Alternately, the activities of MDHAR, POD and SOD in the RNAi plants were also consistent with the results obtained for *osnadk1* (Additional file 4: Figure S4).

### Expression profiles of stress response-related genes in the OE and RNAi plants

**Fig. 9:**
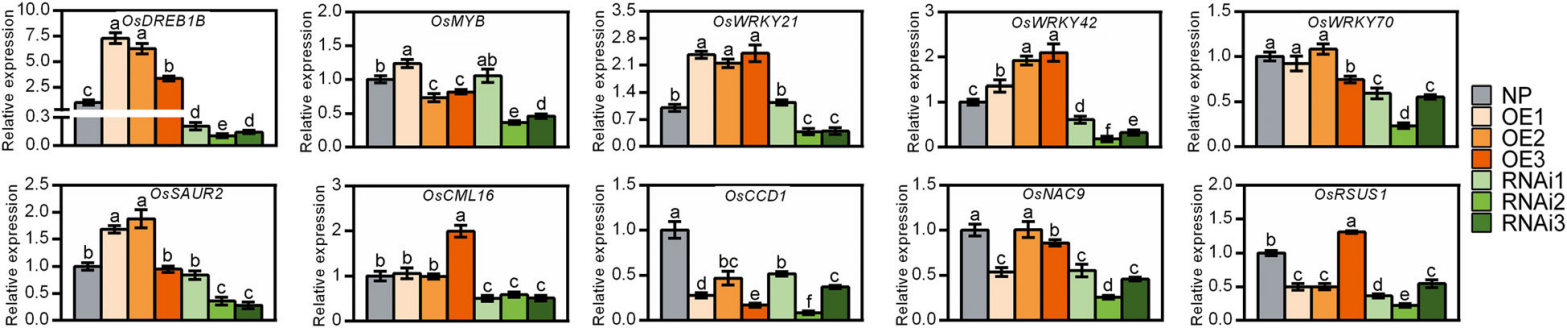
Relative expression levels of genes in transgenic plants detected by qRT-PCR. The gene symbols and primer sequences used for qRT-PCR are listed in Additional file 8: Table S3. Data are means±SD from three independent biological replicates. Bars annotated with different letters represent values that were significantly different by the Tukey method (*p* ≤ 0.05)

Next, the expression profiles of the 10 selected genes as mentioned above were characterized in the transgenic plants by qRT-PCR (Fig. 9). Genes encoding the transcription factors OsDREB1B*,* OsWRKY21, OsWRKY42, and OsSAUR2 all exhibited expression profiles paralleling that of *OsNADK1*. The expression levels of the four genes were upregulated markedly in almost all the OE lines but downregulated in almost all the RNAi lines (Fig. 9). In addition, *OsWRKY70*, *OsCML16* and *OsNAC9* were downregulated in the RNAi plants, whereas the expression profiles of the other genes were not correlated with *OsNADK1* in the transgenic plants compared to WT (Fig. 9). Considering that the expression profile of *OsDREB1B* well paralleled that of *OsNADK1* and that the promoter region of *OsNADK1* contains a non-conservative element that binds DREB transcription factors, we then examined whether OsDREB1B could bind to the *OsNADK1* promoter to feedback regulate *OsNADK1* expression using the dual-luciferase reporter assay. However, as shown in Additional file 5: Figure S5, OsDREB1B could not directly bind to the promoter of *OsNADK1*, implying that OsDREB1B might function as a downstream of OsNADK1 against drought stress in rice.

## Discussion

The rice genome contains four NADK genes, *OsNADK1* to *4*, which exhibit different expression patterns in different tissues and under diverse external conditions [6]. For *OsNADK3*, lack of function leads to dwarfism and sterility [18]. In the present study, we found that OsNADK1 is a cytosolic protein that may play a role in drought-stress tolerance in rice.

### *OsNADK1* is expressed throughout the rice plant and its transcripts can be strongly stimulated by a variety of environmental factors

The qRT-PCR and GUS reporter results showed that *OsNADK1* was expressed in all examined tissues during all examined developmental stages, with a maximum quantity of transcripts produced by the most vigorous organs at every stage (Fig. 1), indicating that *OsNADK1* functioned in the whole plant and in all developmental stages. Unlike the widespread variety of OsNADK1-organ locations, AtNADK1 was expressed only in the roots of *Arabidopsis* [10]. AtNADK1 is also a cytosolic enzyme that likely supplies NADP to cytosolic enzymes and potentially to organelles [10, 39]. We have previously reported that, in addition to the four rice NADKs, there are four wheat NADKs, two of which, namely, TaNADK1 and TaNADK2, which are homologs of OsNADK1 and OsNADK2, respectively, are cytosolic [6, 12]. In contrast, only three NADKs were found in *Arabidopsis*, and only AtNADK1 is a cytosolic enzyme [10]. Apparently, although OsNADK1 was found in the cytosol (Fig. 2), and its lack of function affected the NADP(H) concentrations (Fig. 5c, d), its function might be different from that of AtNADK1 because of their quite dissimilar expression patterns, as discussed above.

OsNADK1 may contribute to stress tolerance in rice as its transcripts, especially in leaves, are strongly stimulated by a number of environmental factors, including cold, heat, dehydration, oxidative stress and ABA, MeJA and SA treatments (Additional file 2: Figure S2). ABA, JA and SA can trigger three antagonistic signalling pathways in plants that participate in stress defence in conjunction with the reactive oxygen species (ROS) metabolic system [40]. Production of ROS is an intrinsic characteristic of plants when they are faced with environment stresses. Plants protect themselves by increasing their antioxidative activity and antioxidant concentrations [41, 42]. Considering the roles of NADKs in ROS production via NADPH oxidase activity [22] and ROS homeostasis in different subcellular compartments of plant cells [30], the present results suggest that OsNADK1 might be involved in the aforementioned hormone signalling pathways in the cytosol by regulating ROS production.

### OsNADK1 activity affects the intracellular redox balance in rice

The *osnadk1* mutant was sensitive to drought and showed a faster water loss rate (Fig. 4). Additionally, the transgenic plants showed similar phenotypes to *osnadk1* after drought (Fig. 8). Together with the observation that the expression level of *OsNADK1* and the total NADK activities in rice plants could be increased by drought (Fig. 4), it could be concluded that OsNADK1 played a role in drought tolerance in rice.

The role of OsNADK1 in drought tolerance might be involved in its functions in maintaining the intracellular redox balance. As the three major intracellular redox couples, NADPH/NADP, ASA/DHA and GSH/GSSG appear to be the most important ones participating in environmental stress responses [31, 32]. NADKs mainly contribute to NADP production in plant cells, and the resultant NADP could be rapidly reduced to NADPH by a series of NADP-dependent dehydrogenases [30, 34–36]. In addition, Mailloux et al. found that NADK can diminishes oxidative damaged caused by environmental stress with enhanced NADPH biosynthesis in *Pseudomonas fluorescens* [33]. Gray and colleagues found that NADK could regulate the size of the NADPH pool and the NADPH/NADP ratio, thereby influencing the intracellular redox balance and oxidative defence system in rat pancreatic β-cells [27]. The mutant of *AtNADK2* is sensitive to drought with a decreased content of NADP(H) in *Arabidopsis* [15, 28, 34–36]. In the present study, we found that the amounts of NADP and the ratio of NADP(H)/NAD(H) were clearly decreased in the *osnadk1* mutant and RNAi lines but increased in the OE lines under normal conditions, whereas the ratios of NADPH/NADP and NAD(P)H/NAD(P) were increased in the *osnadk1* mutant and RNAi lines (Fig. 5; Additional file 4: Figure S4). In addition, the contents of NAD, NADH, NADP and NADPH and their NADH/NAD and NADP(H)/NAD(H) ratios in *osnadk1* were all lower than WT when the plants were subjected to drought (Fig. 5). These results suggest that OsNADK1 participated in the NADP supply and NAD(H)/NADP(H) balance in rice cells. Loss-of-function of OsNADK1 harmed the NADP supply and NAD(H)/NADP(H) balance and decreased the drought tolerance in rice.

Lack of OsNADK1 also harmed the balance of another two redox couples, ASA/DHA and GSH/GSSG, since the ratios of ASA/DHA and GSH/GSSG were substantially decreased in *osnadk1* under both normal growth and drought stress condition (Fig. 5). Although we were unable to find a direct relationship between the contents of GSH and GSSG and the activity of NADK in the transgenic plants, the contents of ASA and DHA were highly consistent with the activity of NADK in these transgenic plants (Additional file 4: Figure S4). Previously, a decrease in the GSH/GSSG value was observed in *atnadk3*, an *Arabidopsis* AtNADK3 knockout mutant that is sensitive to oxidative stress [16]. Another study has also shown that GSH/GSSG ratios may affect the overall redox potential of cells via NAD/NADP metabolism [5]. In addition, Schwanz and colleagues found that the amount of reduction ASA increases in oak trees under drought conditions despite the decrease in total ASA (ASA + DHA) [43]. In the roots of a salt-sensitive tomato cultivar, Shalata and colleagues have found a decreased amount of ASA but an increased level of DHA when the plants are exposed to a salt environment, leading to a smaller ASA/DHA value [44]. Here we found that, although the amount of total ASA was nearly the same among different *OsNADK1* transgenic plant lines, it was much higher in the OE lines than WT and RNAi lines; in contrast, the amount of DHA was lower in the OE lines than the WT and RNAi lines (Additional file 4: Figure S4). Similar results were also found between *osnadk1* and WT (Fig. 5). These results suggested that the amount of ASA and DHA in rice cells might be related to the activity of OsNADK1.

The role of OsNADK1 in the intercellular redox balance of rice may also be indirectly supported by the results obtained from a set of antioxidant enzymes such as DHAR, MDHAR, GR, CAT, APX, POD and SOD (Fig. 5, Additional file 4: Figure S4). These enzymes were discovered as essential components of the Halliwell-Asada cycle and antioxidant system, playing marked roles in ASA and GSH recycling and ROS degradation when plants are responding to environmental stresses [45, 46]. As such, the activities of these enzymes indirectly influence the intracellular redox balance. Furthermore, when plants are subjected to environmental stresses, the activities of these enzymes are often increased. This increment is considered to be a major hallmark of the stress responses of plants [47–51]. Therefore, although the activities of only three enzymes, DHAR, MDHAR and GR, exhibited much higher levels in the *osnadk1* mutant compared with WT under drought and loss of OsNADK1 appeared to weaken the responsive flexibility of the antioxidant enzymes to drought, the marked upregulation of the activities of these enzymes in *osnadk1* under normal growth condition suggested that the absence of OsNADK1 could trigger a stress response in *osnadk1*, possibly by disturbing the intracellular redox balance in the plants.

### The redox-related OsNADK1 action against drought is complicated

Due to the fundamental role of OsNADK1 in the intracellular redox balance and its increased expression in response to various environmental factors, it is reasonable to assume that OsNADK1 activity participates in stress tolerance. As expected, the results obtained from the microarray analysis and the drought-tolerance tests involving *osnadk1* and/or the transgenic plants (Figs. 4, 6 and 8) showed that OsNADK1 activity was related to drought conditions. Judging from the microarray assay results, under both normal growth and drought conditions, hundreds of genes showed altered expression in *osnadk1* compared with WT (Additional file 6: Table S1, Additional file 7: S2), especially the expression of transcription factor genes, e.g., *OsDREB1B* and *OsWRKY* family members. OsDREB1 is a CCAAT-binding transcription factor belonging to the AP2 subfamily. It responds to a number of abiotic/biotic stresses, and its transgenic plants exhibit greater tolerance to various environment stresses [52–56]. Ito and colleagues found that *OsDREB1A* expression does not respond to a large concentration of NaCl and drought conditions, but its overexpressing lines have enhanced tolerance to both conditions [52]. In addition, Ishizaki and colleagues found that when *OsDREB1B* is overexpressed in an upland rice cultivar, the plants have an enhanced tolerance to drought [56]. The expression of *OsDREB1B* is decreased in *osnadk1* and the RNAi transgenic plants but greatly upregulated in OE plants in parallel with OsNADK1 activity (Additional file 6: Table S1; Figs. 7 and 9), showing that OsDREB1B is related to the OsNADK1-mediated stress tolerance. OsDREB1B might participate downstream of the OsNADK1-mediated process during stress tolerance, even though it could not directly bind to the *OsNADK1* promoter (Additional file 5: Figure S5). In fact, many studies have shown that DREBs act as redox-responsive transcription factors in plants, as their activity results in the adjustment of the cytoplasmic redox state [57–59]. For instance, Desikan and colleagues found that the expression of DREB2A is controlled by the cellular redox state [60]. Therefore, it is reasonable to believe that the functions of OsDREB1B in OsNADK1-mediated stress tolerance might also be controlled by the cellular redox state because OsNADK1 activity impacts for the intracellular redox balance in rice, as discussed above.

It has been reported that overexpression of OsDREB1A can increase the amount of free proline under normal and stress conditions [52]. Overexpression of SiARDP, an ABA-responsive DREB-binding protein from foxtail millet, can also enhance the drought and salt tolerance of transgenic *Arabidopsis* plants with greater proline accumulation under drought in the transgenic plants [61]. Overexpression of the buckwheat DRE-binding transcription factor gene *FeDREB1* enhances the drought tolerance of a transgenic *Arabidopsis* line and increases its proline content [62]. Guerrier and colleagues also reported that the proline content may be related to the variation in NADP(H) content controlled by NADK when the calli of soybeans are grown for a short time under aphotic and NaCl conditions [63]. Ruiz and colleagues found a correlation between Ca^2+^/CaM-dependent NAD kinase activity and proline metabolism in green bean when the plants were subjected to cold shock, and later, they found that the relationship is also an adaption to short-term salt stress [64, 65]. These results suggest a relationship between NADK and DREB activities and proline content in plant stress tolerance.

WRKY transcription factors, which contain the conserved WRKYGQK sequence at their N-terminals, are important for the regulation of gene expression, defence responses, and growth regulation [66–70]. In *Arabidopsis*, many WRKY family genes have been found to be redox-sensitive [57]. In rice, at least eleven WRKY transcription factors have been reported to regulate the response of the plant to *Magnaporthe oryzae*, and OsWRKY42 was found to act as a transcriptional repressor, negatively regulating the response of rice to blast fungus by suppressing genes related to JA signalling [71]. In the present study, the expression of a set of WRKY family genes, including *OsWRKY21*, *24*, *28*, *42*, *69*, *70*, and *71,* was affected by the absence of OsNADK1. Among these genes, seven were downregulated in *osnadk1* compared with WT, even under normal growth conditions (Additional file 6: Table S1, Fig. 7), indicating that these WRKY transcription factors might also participate in the OsNADK1-mediated process. However, only *OsWRKY21* and *OsWRKY42* were highly co-expressed with *OsNADK1* (Fig. 9). Recently, Cheng and colleagues reported that *OsWRKY42* RNAi plants enhance the tolerance of rice against *M. grisea*. However, the tolerance is diminished when *WRKY42* is overexpressed [71]. Our results are not consistent with the findings of Cheng and colleagues because we observed an appreciable increase in expression of *OsWRKY21* and *OsWRKY42* in the *OsNADK1*-overexpressing plants but a decrease in the RNAi plants (Fig. 9).

Additionally, the genes *OsCML16* [72], *OsCCD1* [73], *OsNAC9* [74, 75] and *OsRSUS1* [76] have been reported to participate in the resistance of plants to various stresses. In the present study, we found that these genes might also participate in the activities of OsNADK1-mediated drought tolerance, although they did not exhibit a highly consistent co-expression pattern with *OsNADK1* in WT, *osnadk1*, or both transgenic plants (Additional file 6: Table S1, Additional file 2: Figure S2, Figs. 7 and 9).

## Conclusions

In this study, a cytosol-localized NADK, OsNADK1, was cloned and characterized in rice. OsNADK1 affects the intracellular redox balance and functions in drought tolerance of rice. A redox-related mechanism might contribute to the OsNADK1-mediated stress tolerance, possibly affecting the proline content in rice. Considering the fact that overexpression of *OsNADK1* gene enhanced the tolerance of transgenic plants to drought, the results obtained here also suggest that OsNADK1 may act as a candidate for improving drought tolerance of rice by biotechnological approaches.

## Methods

### Characterization of *OsNADK1* expression at different rice developmental stages

The expression profiles of *OsNADK1* during different rice developmental stages and in tissues affected by different environmental factors were examined by real-time PCR (qRT-PCR) with *OsActin1* and *OsUBQ* as the internal controls [77]. Total RNA was extracted from the tissues using RNAiso™ Plus reagent (Takara, Dalian, China) and then treated with RNase-free DNase I (Takara). The concentration and quality of the RNA samples were measured using a NanoDrop 1000 spectrophotometer (Thermo, Waltham, MA, USA). First-strand cDNA was synthesized using EasyScript First-Strand cDNA Synthesis SuperMix kit reagents (TransGen Biotech, Beijing, China). Expression levels of *OsNADK1* were quantitated using UltraSYBR Mixture kit reagents (Kangwei, Beijing, China) and a CFX96 Touch Real-Time PCR Detection system (Bio-Rad, Hercules, California, USA). The primer sequences used are shown in Additional file 8: Table S3. The RT-PCR experiment was carried out in three biological repeats, each with three technical replicates.

### Subcellular location of OsNADK1

The *OsNADK1* coding region was cloned into a modified pUC18 vector between a 35S promoter and a sequence encoding GFP using reagents from a One-Step Directional Cloning kit (Novoprotein, Shanghai, China). The 35S:*OsNADK1*-*GFP* plasmid and control 35S:*GFP* plasmid were separately transfected into rice protoplasts using a polyethylene glycol-calcium-mediated system [78]. After incubation for 16 h at 25 °C with shaking at 40 rpm under aphotic condition, the protoplasts were examined using an A1R confocal microscope (Nikon, Tokyo, Japan). For nuclear staining, the DAPI stock solution was diluted to 300 nM in PBS buffer, and the solution was then added to the W5 solution with protoplasts for 30 min. The samples were examined using an A1R confocal microscope.

### Identification of cis-acting elements in the *OsNADK1* promoter, cloning and transfection of the *OsNADK1* promoter: GUS construct, and GUS staining in transfected rice organs

The *OsNADK1* promoter sequence was obtained from the Joint Genome Institute website (https://phytozome.jgi.doe.gov/pz/portal.html) and analysed with PlantCare (http://bioinformatics.psb.ugent.be/webtools/plantcare/html/) [6]. The promoter sequence was cloned into a pCAMBIA1301 vector (replacing the 35S promoter) to control GUS gene expression using One-Step Directional Cloning kit reagents (Novoprotein). The vector was introduced into rice calli (cv. Nipponbare) by *A. tumefaciens*-mediated transfection as described previously [79]. The tissues and organs at each development stage of the transgenic plants were submerged into a GUS-staining solution and dehydrated under a vacuum for 30 min. The samples were then held at 37 °C for 2 days and decolorized in ethanol before photographing them with a Nikon D90 camera [80].

### Mutant identification and transgenic plant generation

The *osnadk1* mutant line PFG_1A-07609.R and its WT line cv. Dongjin were purchased from Pohang University of Science and Technology, Korea. The homozygotes were identified by PCR as described previously [77]. The homozygous *OsNADK1* knockouts were screened by general PCR with three primers: Left primer (LP), 5′- GAGCCTCTGCACCCTTATTG-3′, Right primer (RP), 5′-TGCTTTTGCAACAGCTTCAG-3′, and T-DNA primer (BP), 5′-AACGCTGATCAATTCCACAG-3′. The T-DNA insertion copies of *osnadk1* were detected by southern blotting with the DIG High Prime DNA Labeling and Detection Starter Kit II as we have described previously [77]. The genomic DNA of WT and *osnadk1* was digested with BamHI and SacI, respectively. The DIG-labelled probes were as follows: 5′ -CTTCTCGTTGGGGTCTTTGC-3′ and 5′ -CATTTCTTGTTTGTGCTGTTCTC-3′. The expression level of *OsNADK1* in the mutant was tested by semi-quantitative PCR and qRT-PCR. The number of cycles was 40 for both experiments. The primers used are listed in Additional file 8: Table S3. Chlorophyll was extracted with 95% ethanol from 0.1 g four-week-old seedlings utilizing the method of Yang et al. [81]. The samples were ground into homogenate and then filtered into a 25-mL brown glass volumetric flask. The extracts were measured at 665 and 649 nm.

To construct the *OsNADK1*-overexpressing transgenic plants, the CDS of *OsNADK1* was cloned into pCAMBIA1301 (modified) under the ubiquitin promoter. The primers used were as follows: forward, 5′ - cgg***GGTACC***ATGTCGCTCGACGAGCTTCC - 3′ (KpnI site in bold/italics, lowercase letters protecting the bases) and reverse, 5′ - ccg***GAGCTC***TCAATCACGCGGGCCGTCG - 3′ (SacI site in bold/italics, lowercase letters protecting the bases). The RNAi vector was constructed by choosing the highly specific products of OsNADK1 as the RNAi sequence. The primers used were as follows: forward, 5′ - ggg***GGTACCACTAGT***CTTGGTGGTGATGGGACTGTT - 3′ (bold/italics are the restriction enzyme sites KpnI and SpeI, lowercase letters are protecting the bases) and reverse, 5′ - ggg***GGATCCGAGCTC***GAATATGCCGTGCTTCCAGAT - 3′ (bold/italics are the restriction enzyme sites BamHI and SacI). The products were linked in the forward and reverse directions into the pTCK303 vector, respectively, to form a hairpin structure. The transformation vectors were introduced into *Agrobacterium tumefaciens* stain EHA105. The calli of rice (cv. Nipponbare) were used for genetic transformation [79]. The expression level of *OsNADK1* in the transgenic lines was detected by qRT-PCR with *OsActin1* and *OsUBQ* as the internal controls. The primers used for the qRT-PCR analysis are listed in Additional file 8: Table S3.

### Characterization of redox couples and enzymatic antioxidant activity

Contents of NAD(P) and NAD(P)H were measured according to the method described by Gibon and Larher [82]. The experimental procedure was carried out according to the protocol supplied with the NAD(H) and NADP(H) measurement kits (KeMing, Suzhou, China). The standard curves were as follows: NAD, y = 0.0416x-0.0139, R^2^ = 0.9986; NADH, y = 0.0351x + 0.0137, R^2^ = 0.9997; NADP, y = 0.0152x + 0.1182, R^2^ = 0.9992; NADPH, y = 0.2486x + 0.2607, R^2^ = 0.9996. The 0.1-g sample was homogenized in 1 mL acid extract (for NAD and NADP) or 1 mL alkaline extract (for NADH and NADPH) on ice. The homogenates were boiled for 5 min, cooled on ice, and then centrifuged at 10,000 g, 4 °C for 10 min. The supernatants were transferred into another clean Eppendorf tube, and after addition of the same volume of alkaline or acid extract for neutralization, the samples were centrifuged at 10,000 g, 4 °C for 10 min. The resultant supernatants were kept on ice for detection. The NAD(P)H can turn 3-(4,5-dimethyl-2-thiazolyl)-2,5-diphenyl tetrazolium bromide (MTT) into formazan by a reduction reaction with the help of phenazine methosulfate (PMS) as a hydrogen carrier. NAD reduced to NADH in the presence of alcohol dehydrogenase, and NADP reduced to NADPH by 6-phosphogluconate dehydrogenase, and then the contents of NADH and NADPH were measured by the MTT method as described above.

Contents of ASA and DHA were determined according to Queval and Noctor [83] as well as Ueda et al. [84]. The assays were performed as follows: 100 mg leaf tissue were ground into powder in liquid nitrogen and then extracted with 1 mL 0.2 M HCl. After centrifugation at 14000 rpm, 4 °C for 10 min, an aliquot of 0.5 mL supernatant was added to 100 μL 0.2 M phosphate buffer pH 5.6 and vortexed. The samples were then neutralized by addition of 400 μL 0.2 M NaOH to adjust the pH to 4–5. To measure the reduced form, ascorbate (ASA), the mixture of 100 μL 0.2 M phosphate buffer pH 5.6, 55 μL H_2_O and 40 μL of the neutralized extracts in the plate wells was measured at A_265_. After the first read, 5 μL ascorbate oxidase (AO, 40 unit mL^− 1^) was added to the mixture followed by continuous reading for 5 min. To measure the oxidized form, dehydroascorbate (DHA), and the total ascorbate, the separate extract aliquots were treated with a DHA-reducing compound such as dithiothreitol (DTT). An aliquot of 0.1 mL neutralized extracts was mixed with 0.14 mL 0.12 M phosphate buffer pH 7.5 and 10 μL 25 mM DTT. After incubation at room temperature for 30 min, triplicate aliquots of 40 μL of the incubated extracts were assayed as described for ASA.

Contents of GSH and GSSG were measured using the GSH and GSSG assay kit (Beyotime, Shanghai, China) based on the 5,5′-dithiobis (2-nitrobenzoic acid) (DTNB)-GR recycling assay as described by Rahman et al. [85]. The samples were ground into powder in liquid nitrogen. Every 10 mg sample was added to 30 μL protein removal reagent M, vortexed, and supplemented with 70 μL reagent M for homogenization at 4 °C for 10 min. After centrifugation at 10,000 g, 4 °C for 10 min, the supernatants were used for total GSH measurements. Ten microliters of the sample extracts and reagent M was added to 150 μL total GSH assay liquid (glutathione reductase, DTNB stock solution and total GSH assay buffer). After incubation for 5 min at 25 °C or room temperature, the reaction was started by addition of 50 μL NADPH (0.16 mg mL^− 1^) and then monitored at 412 nm. GSSG was measured as follows: 100 μL extracts was added to 5 μL GSH removal auxiliary liquid, vortexed, and supplemented with 1 μL GSH removal reagent, 25 °C for 60 min. The total GSH assay method was then repeated.

Determination of the activities of antioxidant system enzymes, namely, dehydroascorbate reductase (DHAR, EC 1.8.5.1), monodehydroascorbate reductase (MDHAR, EC 1.6.5.4), glutathione reductase (GR, EC 1.8.1.7), catalase (CAT, EC 1.11.1.6), ascorbate peroxidase (APX, EC 1.11.1.11), peroxidase (POD, EC 1.11.1.7) and superoxide dismutase (SOD, EC 1.15.1.1), was performed as described by Duan et al. [86] and Noctor et al. [87]. Leaf samples (0.5 g) were homogenized in sodium phosphate buffer (0.05 M, pH 7.0). The extracts were centrifuged at 10,000 g, 4 °C for 20 min, and the supernatants were used for the enzyme assays. DHAR activity was measured in 0.1 M phosphate buffer (1 mL) containing 0.2 mM DHA, 2.5 mM GSH, 1 mM EDTA (pH 7.0) and 20 μL extracts, monitored at 265 nm for 3 min. MDHAR activity was assayed in 50 mM HEPES buffer (pH 7.6, 1 mL) containing 0.25 mM NADPH, 2.5 mM ascorbate and 50 μL extracts. The reaction was then started by addition of 5 μL of 0.4 units AO at 340 nm for 3 min. GR activity was measured in 0.1 M phosphate buffer (pH 7.5, 1 mL) containing 0.1 mM NADPH, 1 mM EDTA and 100 μL extracts. The reaction was started by addition of 10 μL of 50 mM GSSG and monitored at 340 nm for 3 min. CAT activity was measured in the mixture (3 mL) containing 15 mM sodium phosphate buffer (pH 7.0), 10 mM H_2_O_2_ and 0.1 mL extracts, and the absorbance was recorded at 240 nm for 3 min. APX activity was measured in the 50 mM potassium phosphate buffer (pH 7.0) containing 1.0 mM EDTA, 0.3 mM ASA and 50 μL extracts. The reaction was started by addition of 20 μL of 9 mM H_2_O_2_ and monitored at 290 nm for 3 min. POD activity was measured in the 50 mM potassium phosphate buffer (pH 7.0) containing 20 mM guaiacol and 10 μL extracts. The reaction was started by addition of 10 μL H_2_O_2_ and monitored at 470 nm for 3 min. SOD activity was measured in the 50 mM potassium phosphate buffer (pH 7.8) containing 13.37 mM DL-methionine, 77.12 μM nitro-blue tetrazolium, 0.1 mM EDTA, and 2.0 μM riboflavin. Then, 0.1 mL of the extracts and 3.9 mL buffer were added to two tubes. One tube was placed under photic condition for 15 min, and the other was exposed to aphotic condition as the control. In addition, 0.1 mL potassium phosphate buffer and 3.9 mL buffer were added to a tube as the photic control.

The total NADK activity was measured using a NADK-assay kit (KeMing, Suzhou, China). Leaf samples (0.1 g) were homogenized in 1 mL extracts on ice. The extracts were centrifuged at 8000 g, 4 °C for 10 min, and the resultant supernatants were used for the enzyme assays. Twenty microliters of the extracts were mixed with 80 μL working solution I and then incubated at 25 °C for 15 min, boiled for 2 min, and cooled on ice. After centrifugation at 10,000 g, 25 °C for 10 min, 25 μL supernatants were added to 110 μL working solution II and 110 μL working solution III. After mixing, NADP production was measured at 600 nm for 3 min. The activity of NADK was calculated as follows: NADK (nmol min^− 1^ mg^− 1^ protein) = 812*(∆A - 0.004)*V/(V*Cpr*T), where ∆A denotes the change in absorbance, V denotes the volume of the extract in the reaction, Cpr is the content of protein, and T is the reaction time.

### Assessment of stress tolerance and proline contents

Four-week-old wild-type (WT, cv. Dongjin or cv. Nipponbare), *osnadk1*, and OE and RNAi transgenic seedlings grown in soil (Matrix: soil = 1:1) were exposed to drought conditions. The WT and *osnadk*1 were planted in pots with a size of 25*15*15 cm, and the WT and OE and RNAi transgenic seedlings were planted in pots with a size of 45*25*20 cm. The greenhouse was kept at 26 °C with a 12/12-h day-night cycle. When the seedlings had been living without water for 10 days (moderate drought period, physiological measurements were made) or for 2 weeks (severe drought treatment), the plants were irrigated respectively and then the survival rates measured. After the drought-stress treatments and water recovery, the plants that still had green and healthy young leaves were considered to have survived. To measure the survival rates, at least 25 plants from each line, each with three biological repeats, were included in the experiments.

Proline content was measured as described previously [88]. Samples were boiled in a 3% (w/v) sulfosalicylic acid solution for 10 min. After cooling to room temperature, the extracts were filtered separately through a Whatman 9-cm filter, and the filtered liquid was used for measurements of proline as reported.

### Microarray gene expression assay

GeneChip rice-genome arrays (Affymetrix, Santa Clara, CA, USA) were used to characterize global gene expression and were performed by Shanghai Bohao Corporation (Shanghai, China). Rice WT (*O. sativa* ssp. *japonica* cv. Dongjin) and *osnadk1* mutant seeds were used for the experiment. The sample treatments and experimental processes have been described previously [77]. Only genes with transcript levels that had increased or decreased more than two-fold with associated *p*-values < 0.05 were used in the Venn diagrams and pie charts and further analysed.

### Transient transcriptional activity assay using the dual-luciferase system

The promoter of *OsNADK1* was used for the transient transcriptional activity assay as a reporter and cloned into the PGL3 dual luciferase vector [89] instead of the 2 × 35S promoter to control Rluc (Renilla Luciferase). The primers used were as follows: forward, 5′ - ccgGAGCTCGGGCAAGTTCAAATCAGTGG - 3′ (SacI site in bold/italics, lowercase letters are protecting the bases) and reverse, 5′ - ctaGCTAGCGGCGGCGCGATCCGGCGGTT - 3′ (NheI site in bold/italics, lowercase letters are protecting the bases). The OsDREB1B-eGFP construction was used as the effector and empty eGFP vector as the control effector (Additional file 5: Figure S5a). The CDS of *OsDREB1B* was cloned into the pUC18 (modified) vector using one-step directional cloning kits (Novoprotein, Shanghai, China) between the 35S promoter and GFP protein. The primers for OsDREB1B-eGFP construction were as follows: forward, 5′ - GTCGACTCTAGA***GGATCC***ATGGAGGTGGAGGAGGC - 3′ and reverse, 5′ - CTTGCTCACCAT***GGATCC***GTAGCTCCAGAGCGGCAT - 3′ (BamH1 site in bold/italics). The two effectors and one reporter were then co-transformed into rice protoplasts, respectively, using the PEG-Ca^2+^ mediated system [78, 89–91]. The transformational protoplasts were gently shaken at 25 °C in the dark. After incubation at 16 h, the protoplasts were transferred to a 1.5-ml Eppendorf tube and centrifuged at 1300 rpm for 5 min. The pellets were resuspended in 20 μL lysis buffer (0.1 M potassium phosphate, 1 mM DTT, pH 7.8). The cells were then frozen and thawed twice and centrifuged at 12,000 rpm, 4 °C for 10 min. The resultant supernatants were transferred into another clean Eppendorf tube to detect the luciferase activity using the Dual-Luciferase® Reporter Assay System (Promega, Madison, Wisconsin, America). For each plasmid combination, six independent transformations were performed.

### Statistical analyses

SPSS 19 software (SPSS, Inc., Chicago, IL) was used for the statistical analyses. Differences were considered significantly at *p* ≤ 0.05 based on the Tukey method or one-way ANOVA.

## Supplementary information

**Additional file 1 **: **Figure S1.** The promoter sequence of *OsNADK1* and its cis-element analysis. The cis-elements were scanned and identified by the PlantCARE programme (http://bioinformatics.psb.ugent.be/webtools/plantcare/html/) with the 2000-bp upstream nucleotide sequence of the *OsNADK1* CDS (coding sequence).

**Additional file 2 **: **Figure S2.** Analyses of *OsNADK1* expression in response to abiotic stresses and hormonal treatments. Relative expression of *OsNADK1* under different abiotic stresses and hormonal treatments, including ABA (100 μM), MeJA (100 μM), SA (0.5 mM), cold (4 °C), heat (40 °C), oxidative stress (30 μM MV), salt stress (200 mM NaCl) and dehydration stress (20% PEG-6000) after 12 h and 24 h of treatment were detected by qRT-PCR. Two-week-old seedlings were used for the analysis. Different letters above the bars represent significant differences by the Tukey method (*p* ≤ 0.05).

**Additional file 3 **: **Figure S3** Southern blot analysis of the T-DNA insertion. M, marker; L1 and L2, WT plants (cv. Dongjin); L3 and L4, *osnadk1* mutant plants; L5, positive control. L1 and L3, *Bam*HI single enzyme digestion; L2 and L4, *Sac*I single enzyme digestion.

**Additional file 4 **: **Figure S4.** Intracellular redox status, activities of antioxidant enzymes and proline content of NP (cv. Nipponbare), *OsNADK1*-overexpression (OE) and RNA interference (RNAi) plants under normal conditions. (a-d, i-k, m-o) The contents of NAD and NADH; NADP and NADPH; GSSG, GSH and (GSSG+GSH); ASA, DHA and (ASA + DHA), respectively. (e-h, j, p) The ratios of NADH/NAD, NADPH/NADP, NADP(H)/NAD(H), NAD(P)H/NAD(P), GSH/GSSG, and ASA/DHA, respectively. (q-w) The enzyme activities of DHAR, MDHAR, GR, CAT, APX, POD and SOD, respectively. All data are the means ± SD and are representative of similar results from three independent experiments. Different letters above the bars represent significant differences by the Tukey method (*p* ≤ 0.05).

**Additional file 5 **: **Figure S5.** Dual luciferase reporter assay system analysis of transcriptional activity. (a) Diagrammatic drawings of the effector and reporter plasmids used in the transcriptional activation in rice protoplasts. RLUC, renilla luciferase; FLUC, firefly luciferase. (b) The *OsNADK1*_*Pro*_: *RLUC* - 2 × 35S: *FLUC* reporter vector was transiently expressed in rice protoplasts together with the control vector (35S: GFP) or OsDREB1B effector, respectively. Data are means±SD from 6 independent biological replicates. Bars annotated with different letters represent values that were significantly different (*p* ≤ 0.05) according to one-way ANOVA.

**Additional file 6 **: **Table S1.** Differentially expressed gene groups in the *osnadk1* mutant compared with WT under normal growth conditions.

**Additional file 7 **: **Table S2.** Differentially expressed gene groups in the *osnadk1* mutant compared with WT under drought stress conditions.

**Additional file 8 **: **Table S3.** The primer sequences used in the study.

## Data Availability

The data used and analysed during the current study are available and shared at NCBI under accession number GSE93917 (microarray).

## Abbreviations

ABA: abscisic acid
APX: ascorbate peroxidase
ASA: ascorbate
CAT: catalase
DHA: dehydroascorbate
DHAR: dehydroascorbate reductase
Fluc: firefly luciferase
GFP: green fluorescent protein
GR: glutathione reductase
GSH: reduce glutathione
GSSG: oxidized glutathione
GUS: β-glucuronidase
MDHAR: Monodehydroascorbate reductase
MeJA: methyl jasmonic acid
MV: methyl viologen
NADK: NAD kinase
OE: overexpressing
POD: peroxidase
qRT-PCR: quantitative real-time RT-PCR
Rluc: renilla luciferase
RNAi: RNA interference
SA: salicylic acid
SOD: superoxide dismutase
WT: wild-type
