# Supplementary material for: The NAD kinase OsNADK1 affects the intracellular redox balance and enhances the tolerance of rice to drought
Source: BMC Plant Biol. 2020 Jan 7;20:11. doi: 10.1186/s12870-019-2234-8 (PMC6947874; doi:10.1186/s12870-019-2234-8)
Supplement: Supplementary file 1 — Additional file 1 : Figure S1. The promoter sequence of OsNADK1 and its cis-element analysis. The cis-elements were scanned and identified by the PlantCARE programme (http://bioinformatics.psb.ugent.be/webtools/plantcare/html/) with the 2000-bp upstream nucleotide sequence of the OsNADK1 CDS (coding sequence). [file 12870_2019_2234_MOESM1_ESM.pdf]

The *OsNADK1*'s promoter sequence and cis-element analysis

```
         10        20        30        40        50        60        70
....|....|....|....|....|....|....|....|....|....|....|....|....|....|
GGGCAAGTTCAAATCAGTGGAAATGTAGCTTCTTTTAGCGGTTCATGTCATTTGACATTTGACGAATACT
                                                         CGTCA-motif
         80        90       100       110       120       130       140
....|....|....|....|....|....|....|....|....|....|....|....|....|....|
CCCAGCTTCACTGATGAATTTTTCAGATAACGACTGAGATGTATACTTGTACGTTTGGTGGCTACCTAAT
                         TGA-element
        150       160       170       180       190       200       210
....|....|....|....|....|....|....|....|....|....|....|....|....|....|
CCAACGCTAGTTCAGAATGAAAGCAACTTTGAGTGCCCCAAAATACAAAGGCAAGTCACTGTACTCGTCA
                                                            CGTCA-motif
        220       230       240       250       260       270       280
....|....|....|....|....|....|....|....|....|....|....|....|....|....|
AGGGTATCTTTATACACGGAATATTGGTCGAGACAAGAGAATTCTAAAGCTTCAGATCCGATGCAAATCT
        290       300       310       320       330       340       350
....|....|....|....|....|....|....|....|....|....|....|....|....|....|
AACATAAGCCAACGAAATATACTTACTACGCAGCTATGTAAAAATGTTGTCTCTCATCTTGGAACATTTT
        360       370       380       390       400       410       420
....|....|....|....|....|....|....|....|....|....|....|....|....|....|
TTTTCCGCGAACGCGCAAAAAAATCGCACGTCAATATATTAGAAGAAAAGAGTTATAGTTACACAACACA
                        CGTCA-motif
        430       440       450       460       470       480       490
....|....|....|....|....|....|....|....|....|....|....|....|....|....|
ATTAGCTAGACCGGCTTATGCCAGGAGAAGAGAGAAAAAGTAAAATAAAAAGCTGAAGCTTATAAAACTA
        500       510       520       530       540       550       560
....|....|....|....|....|....|....|....|....|....|....|....|....|....|
CGGCAGCTAAACACAAACTCCAAGTAGCAGGAAGGGCCCGAGCCACGCTACACTCCCAACAAGTCTCCAA
        570       580       590       600       610       620       630
....|....|....|....|....|....|....|....|....|....|....|....|....|....|
AAGCGGCAATGCTAGTTAAAGACCACGTATGACCCTCTGAGTGGATGGATTTTCAGAACCACTGAGATTG
                        ABRE
        640       650       660       670       680       690       700
....|....|....|....|....|....|....|....|....|....|....|....|....|....|
AAGAGAGCGCAGAGTCGAAAACCCTAGTGTTTCGTTCCTTCCACAACTGCCAAGCGATCAAGAGCACTAG
                                            MBS
        710       720       730       740       750       760       770
....|....|....|....|....|....|....|....|....|....|....|....|....|....|
GAAGTCAAAGCCAGCATGGAGATGCTCAACTAAGCAGGCCCTGAAGGACAGCCACCAATTCTGGAACCAG
        780       790       800       810       820       830       840
....|....|....|....|....|....|....|....|....|....|....|....|....|....|
CTGCCATGGGGAAGAGATAGCAGCCCAGCCAGGGAGGCGATAGCACACGCAACCAGACCTGCCGAGCAAA
```

```
               850       860       870       880       890       900       910
....|....|....|....|....|....|....|....|....|....|....|....|....|....|
CACATAGTCGAGGAGGATGTGTTTCACCGTCTCAAGATCCTAAGGCCATTCCCAACCCATGACACTAGAC
               920       930       940       950       960       970       980
....|....|....|....|....|....|....|....|....|....|....|....|....|....|
ATAGTTTCCATAAACTCCACATCATCAAGAAACTAGTACTAGACACTACTCTTCCAATGCAAATACCACT
               990      1000      1010      1020      1030      1040      1050
....|....|....|....|....|....|....|....|....|....|....|....|....|....|
ATTCCATACTTAGATTTAATGCTATTTATCTCACATGATGCCTTGGATATTGTGTAGAAACCATTGTTTC
                                                            ARE
              1060      1070      1080      1090      1100      1110      1120
....|....|....|....|....|....|....|....|....|....|....|....|....|....|
CTTCTCTTTTCTCATTTATTCACTTGCCACATCATTTTTCATCCTAGGTGGTAGCTTATTTAATGCTATA
              1130      1140      1150      1160      1170      1180      1190
....|....|....|....|....|....|....|....|....|....|....|....|....|....|
GACACTATCCTAGTCATTGGGTTGGAAATGGCCTAAACGCAAAAGGGGCACGCCGAGTGACTATCGATTC
                                          P-box
              1200      1210      1220      1230      1240      1250      1260
....|....|....|....|....|....|....|....|....|....|....|....|....|....|
CACGCTGCTGAAATGCAAACCAAAGGATTCCGCAAAGTACTATAAAATATGTTTACCGGTTTTAATGATT
                   ARE
              1270      1280      1290      1300      1310      1320      1330
....|....|....|....|....|....|....|....|....|....|....|....|....|....|
TTGTAGTCCTTAAAAAGATATTAGAGCTACAAGTTTTTCTAGGATAATTTTAAGAAAAATAGTGTAAAAT
              1340      1350      1360      1370      1380      1390      1400
....|....|....|....|....|....|....|....|....|....|....|....|....|....|
ATAAGAATCTCGAGGTACGGCTCTCTCCTGCTATAAATACCTATAGAGAAGAGGTAACATTACCCTTTTT
              1410      1420      1430      1440      1450      1460      1470
....|....|....|....|....|....|....|....|....|....|....|....|....|....|
TTTCTTATATGAAATCAAACAAGATATCTACTGAAACAACTGGTGTTTAAGATAATATTAAGTTATAAAA
                                           MBS                       HSE
              1480      1490      1500      1510      1520      1530      1540
....|....|....|....|....|....|....|....|....|....|....|....|....|....|
TTTCAAAATTGAAATAATAACTAACCAAGTGACAGAAATATTCTGGTATATGATGGGATGAAAACGGGAA
  ERE
              1550      1560      1570      1580      1590      1600      1610
....|....|....|....|....|....|....|....|....|....|....|....|....|....|
CGGGAAAAGGAAAGGGAACAGACCCACCAACCAACCAACCAAACAAACCGGTTTAGCTTGCTGCTTGCCG
              1620      1630      1640      1650      1660      1670      1680
....|....|....|....|....|....|....|....|....|....|....|....|....|....|
GCAGCAGTAACCACGAAAGCGCTCGGCTCCACACGCCACGCACACCCCACACCCCAACCCAAAGCGGCAT
                                         ABRE
```

```
          1690      1700      1710      1720      1730      1740      1750
....|....|....|....|....|....|....|....|....|....|....|....|....|....|
CAGCGCACGCGTCAGTCGTAGCCTCACTGCGACCGGGACCCACCCACCCCCTGGCCCCACCTGTCAGCCC
       ABRE   CGTCA-motif
          1760      1770      1780      1790      1800      1810      1820
....|....|....|....|....|....|....|....|....|....|....|....|....|....|
CCCGTTGGTGTCAACTCCCGGTGGCCTCCTGCTTCGCCGATAAATAACCGAATCGGGGGGAGGAGGTGGA
          1830      1840      1850      1860      1870      1880      1890
....|....|....|....|....|....|....|....|....|....|....|....|....|....|
GGTGGTGCTCCTCCTCCTCAACAGCGTCGCCTCGCGTCGGCGTCGCCTCTCACCACCCAACCACCAGTCC
          1900      1910      1920      1930      1940      1950      1960
....|....|....|....|....|....|....|....|....|....|....|....|....|....|
ACCACCCACCTCTCTCCCTCCCCTCCCCTCCCCAAACCCAACCCGAGCCGAGGCCGGAATTTCCTTTTTG
           1970       1980       1990       2000 -1
....|....|....|....|....|....|....|....|....|....
GTGCGACGACGACGAGGTGAGGGTGAGCCAACCGCCGGATCGCGCCGCC（ATG:Start condon for
OsNADK1）
```

Cis-elements analysis in OsNADK1's promoter

Plantcare element	Sequence	Position	Species	Function
CGTCA-motif	CGTCA	-320	*Hordeum vulgare*	involved in the MeJA-responsiveness
	CGTCA	-1631	*Hordeum vulgare*	involved in the MeJA-responsiveness
	TCAAG	-1802	*Hordeum vulgare*	involved in the MeJA-responsiveness
	TGACG	-1950	*Hordeum vulgare*	involved in the MeJA-responsiveness
ABRE	CGCACGCGTC	-326	*Hordeum vulgare*	involved in the abscisic acid responsiveness
	GCCACGCACA	-365	*Hordeum vulgare*	involved in the abscisic acid responsiveness
	CACGTA	-1426	*Arabidopsis thaliana*	involved in the abscisic acid responsiveness
ERE	ATTTCAAAA	-540	*Dianthus caryophyllus*	ethylene-responsive element
HSE	ATAAAATTTC	-545	*Brassica oleracea*	involved in heat stress responsiveness
MBS	CAACTG	-573	*Arabidopsis thaliana*	involved in drought-inducibility
	CAACTG	-1336	*Arabidopsis thaliana*	involved in drought-inducibility
ARE	AAACCA	-803	*Zea mays*	cis-acting regulatory element essential for the anaerobic induction
	AAACCA	-972	*Zea mays*	cis-acting regulatory element essential for the anaerobic induction
p-box	CAAAAGG	-850	*Oryza sativa*	gibberellin-responsive element
TGA-element	AACGAC	-1911	*Brassica oleracea*	auxin-responsive element
