# Supplementary figures and images for: The NAD kinase OsNADK1 affects the intracellular redox balance and enhances the tolerance of rice to drought
Source: BMC Plant Biol. 2020 Jan 7;20:11. doi: 10.1186/s12870-019-2234-8 (PMC6947874; doi:10.1186/s12870-019-2234-8)

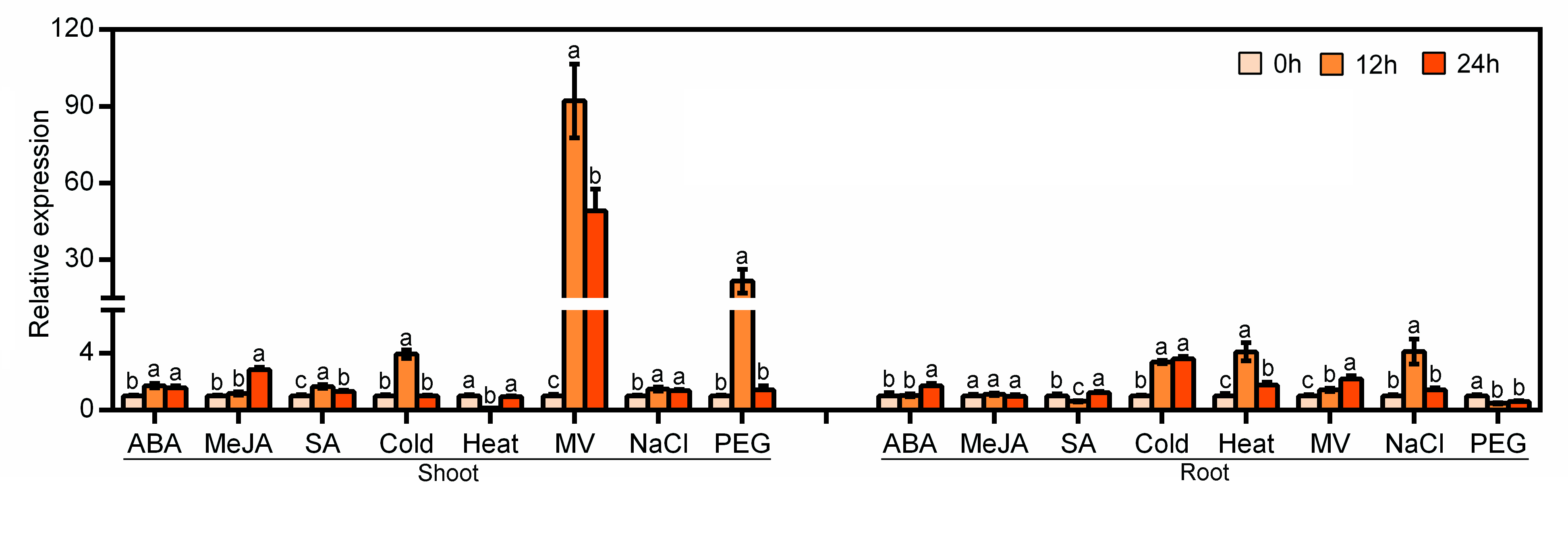

Supplement: Supplementary file 2 — Additional file 2 : Figure S2. Analyses of OsNADK1 expression in response to abiotic stresses and hormonal treatments. Relative expression of OsNADK1 under different abiotic stresses and hormonal treatments, including ABA (100 μM), MeJA (100 μM), SA (0.5 mM), cold (4 °C), heat (40 °C), oxidative stress (30 μM MV), salt stress (200 mM NaCl) and dehydration stress (20% PEG-6000) after 12 h and 24 h of treatment were detected by qRT-PCR. Two-week-old seedlings were used for the analysis. Different letters above the bars represent significant differences by the Tukey method (p ≤ 0.05). [file 12870_2019_2234_MOESM2_ESM.jpg]

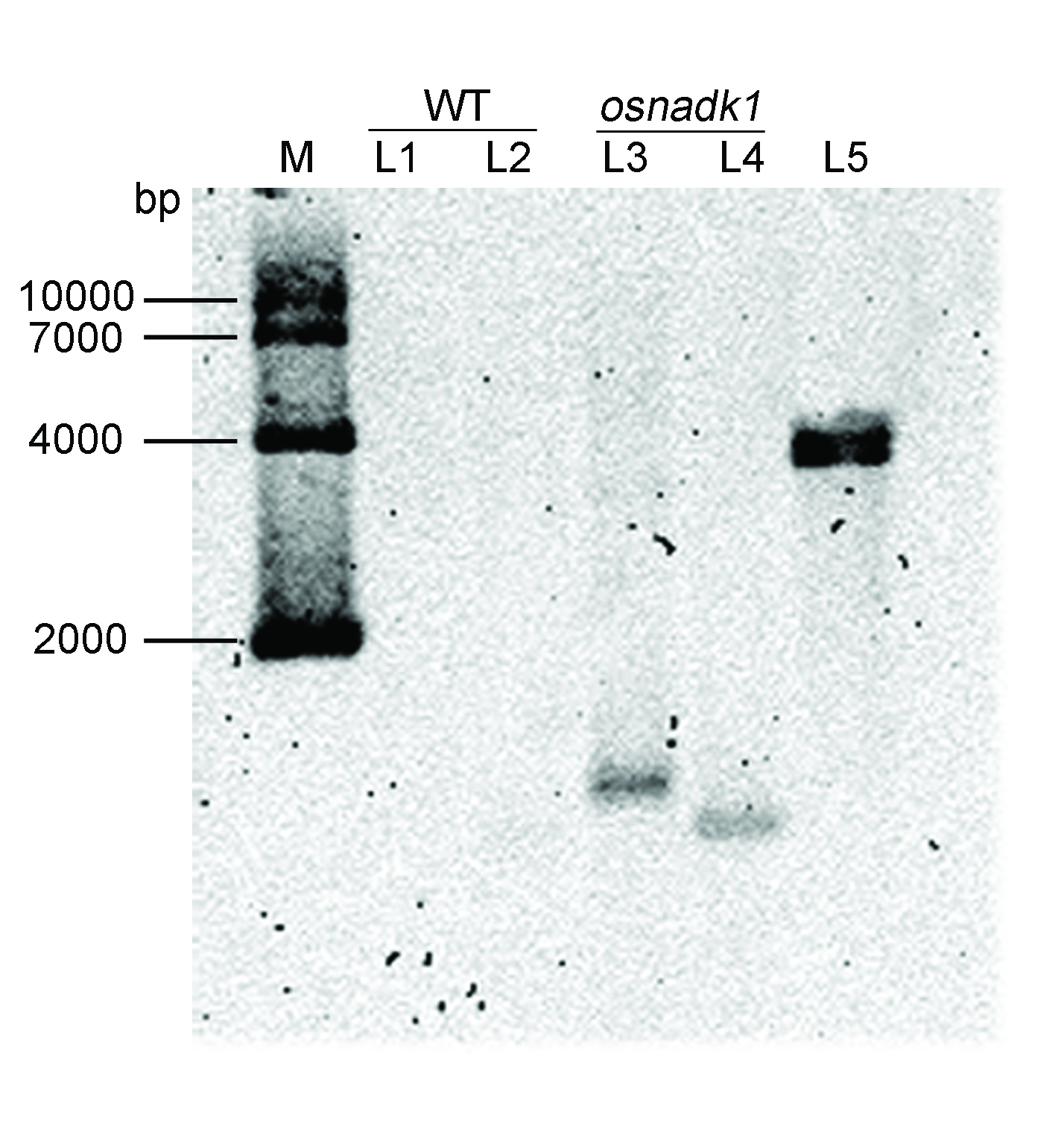

Supplement: Supplementary file 3 — Additional file 3 : Figure S3 Southern blot analysis of the T-DNA insertion. M, marker; L1 and L2, WT plants (cv. Dongjin); L3 and L4, osnadk1 mutant plants; L5, positive control. L1 and L3, BamHI single enzyme digestion; L2 and L4, SacI single enzyme digestion. [file 12870_2019_2234_MOESM3_ESM.jpg]

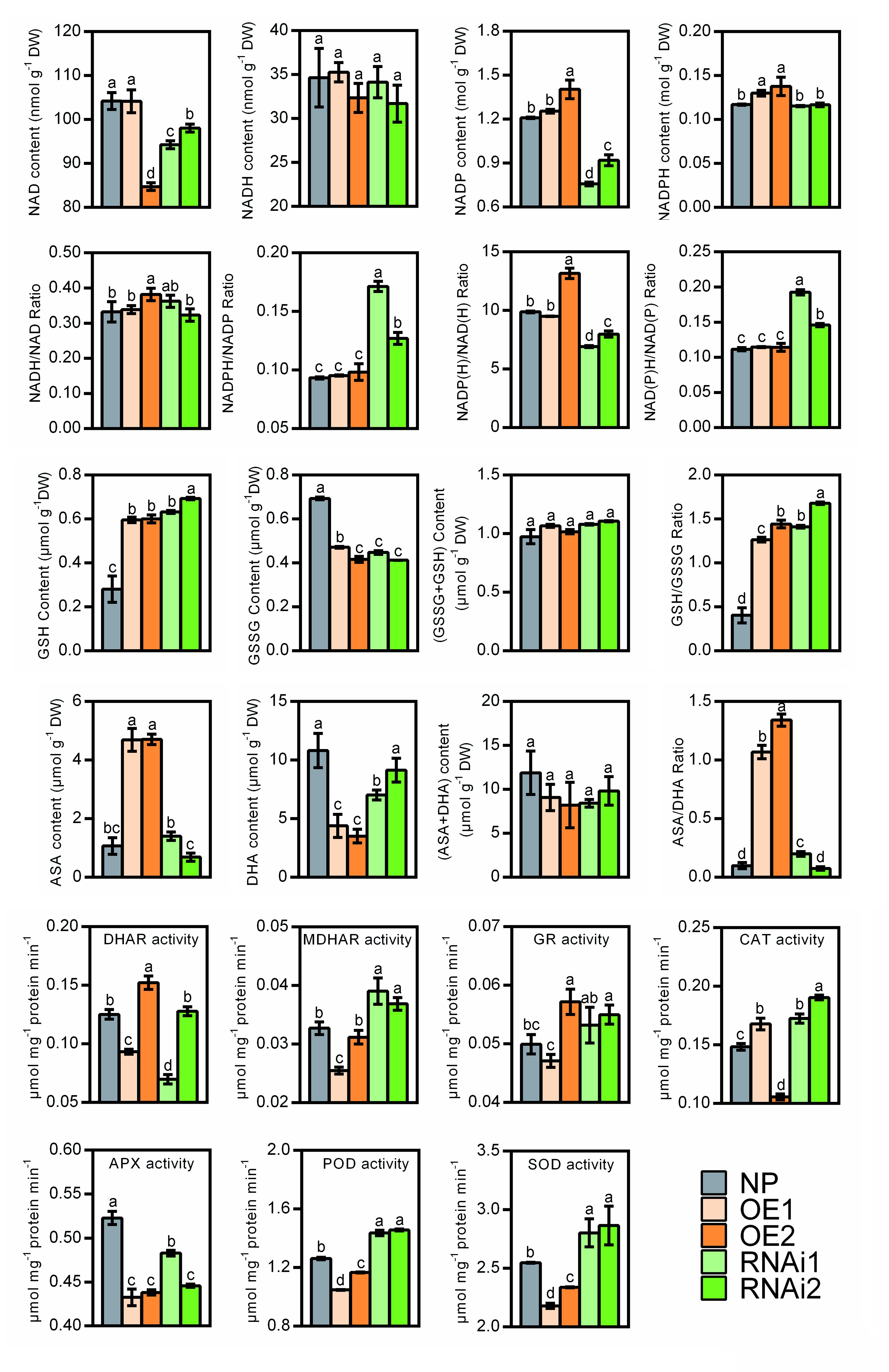

Supplement: Supplementary file 4 — Additional file 4 : Figure S4. Intracellular redox status, activities of antioxidant enzymes and proline content of NP (cv. Nipponbare), OsNADK1-overexpression (OE) and RNA interference (RNAi) plants under normal conditions. (a-d, i-k, m-o) The contents of NAD and NADH; NADP and NADPH; GSSG, GSH and (GSSG+GSH); ASA, DHA and (ASA + DHA), respectively. (e-h, j, p) The ratios of NADH/NAD, NADPH/NADP, NADP(H)/NAD(H), NAD(P)H/NAD(P), GSH/GSSG, and ASA/DHA, respectively. (q-w) The enzyme activities of DHAR, MDHAR, GR, CAT, APX, POD and SOD, respectively. All data are the means ± SD and are representative of similar results from three independent experiments. Different letters above the bars represent significant differences by the Tukey method (p ≤ 0.05). [file 12870_2019_2234_MOESM4_ESM.jpg]

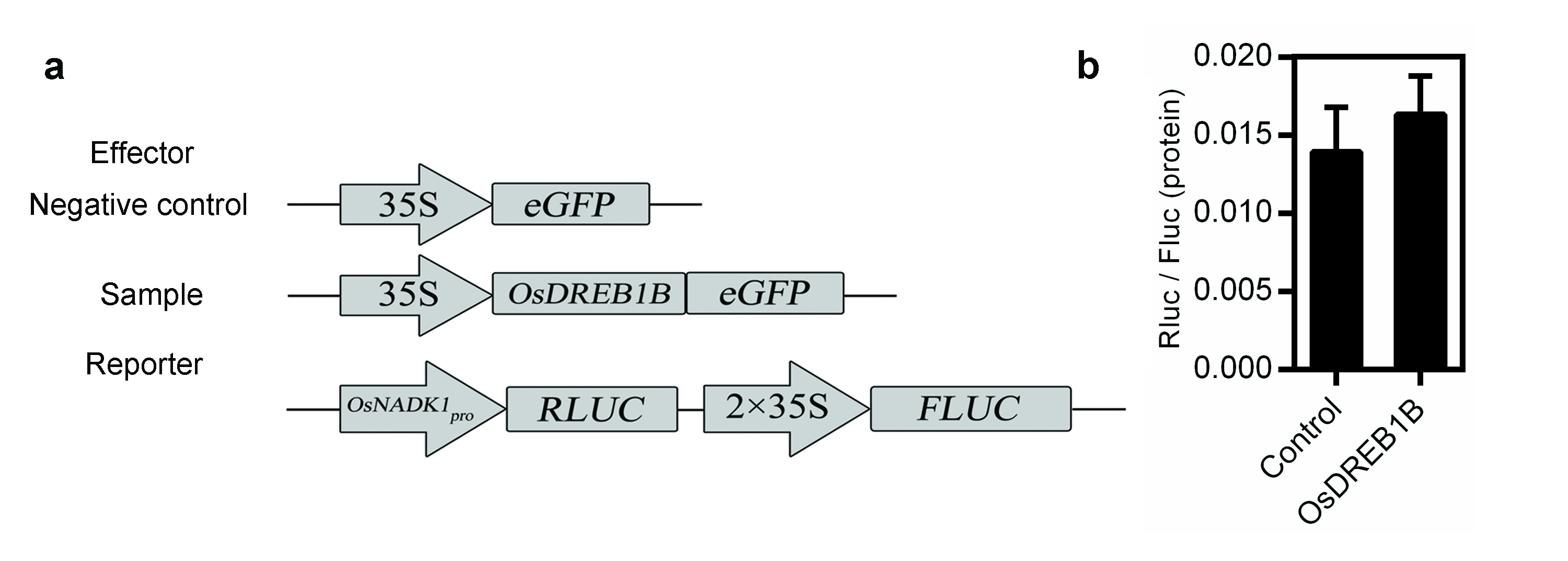

Supplement: Supplementary file 5 — Additional file 5 : Figure S5. Dual luciferase reporter assay system analysis of transcriptional activity. (a) Diagrammatic drawings of the effector and reporter plasmids used in the transcriptional activation in rice protoplasts. RLUC, renilla luciferase; FLUC, firefly luciferase. (b) The OsNADK1Pro: RLUC - 2 × 35S: FLUC reporter vector was transiently expressed in rice protoplasts together with the control vector (35S: GFP) or OsDREB1B effector, respectively. Data are means±SD from 6 independent biological replicates. Bars annotated with different letters represent values that were significantly different (p ≤ 0.05) according to one-way ANOVA. [file 12870_2019_2234_MOESM5_ESM.jpg]
