# Supplementary material for: The NAD kinase OsNADK1 affects the intracellular redox balance and enhances the tolerance of rice to drought
Source: BMC Plant Biol. 2020 Jan 7;20:11. doi: 10.1186/s12870-019-2234-8 (PMC6947874; doi:10.1186/s12870-019-2234-8)
Supplement: Supplementary file 8 — Additional file 8 : Table S3. The primer sequences used in the study. [file 12870_2019_2234_MOESM8_ESM.doc]

Table S3 The primer sequences used in the study.

| Gene Name | Gene_Symbol | Primer Sequences | Amplicon Length |
| --- | --- | --- | --- |
| For real-time PCR | | | |
| *OsActin1* | Os03g0718100 | F: GTGGTCGCCCCTCCTGAAAG | 170bp |
|  |  | R: GGCTTAGCATTCTTGGGTCCG |  |
| *OsUBQ* | Os02g0634800 | F: CCGTTTGTAGAGCCATAATTGCA | 76bp |
|  |  | R: AGGTTGCCTGAGTCACAGTTAAGTG |  |
| *OsNADK1* | Os01g0957000 | F: GATGGGACTGTTTTATGGGCT | 181bp |
|  |  | R: CATGGCACTGTAGACGGTTTC |  |
| *OsDREB1B* | Os09g0522000 | F: AGTAGGCAATGAGACTGAGGATG | 87bp |
|  |  | R: ATCAGATGGAATCACAAAAGGAG |  |
| *OsMYB* | Os01g0298400 | F: TGCCACTCACCTGACCCGT | 102bp |
|  |  | R: GGTCATCAGGTGGTGGTTCG |  |
| *OsWRKY21* | Os01g0821600 | F: GAACTAATGCCACCTGCCGT | 244bp |
|  |  | R: CATTCAAGAACTCCCACACCG |  |
| *OsWRKY42* | Os02g0462800 | F: ATGCAGTCTGCTTCAGATTATGCT | 101bp |
|  |  | R: GACGCCTTCCGTTTTTTCTTG |  |
| *OsWRKY70* | Os05g0474800 | F: ACGGGAGCGTCTTACTCTTACAC | 95bp |
|  |  | R: TCGTCTCTCGGCTCATCCTT |  |
| *OsSAUR2* | Os01g0768333 | F: CAAGGCGAACCTCATCTGTAGC | 99bp |
|  |  | R: CGGGCAGAAATGGAATCAGA |  |
| *OsCML16* | Os01g0135700 | F: AGACGGAGTAAAACAGTGTCAGGT | 203bp |
|  |  | R: AGTAGCATACATCAGCAGATCGGT |  |
| *OsCCD1* | Os06g0683400 | F: GCCTCACAGTTCCTCTTTCATTAG | 111bp |
|  |  | R: CCCTTGATGAATCCTGATGCTAT |  |
| *OsNAC9* | Os03g0815100 | F: TGGTGCCCAAGAAAGAATCG | 175bp |
|  |  | R: GCGAAGAGCGACGAGTAGAAG |  |
| *OsRSUS1* | Os03g0401300 | F: GCCGCTACCTTGAGATGCTGT | 157bp |
|  |  | R: GAAACAAAGCAAAGAAACTCCAATG |  |
| **vector construction** | | | |
| Gene Name | Primer Sequences | | Application |
| OsNADK1:: GFP | F:GTCGACTCTAGAGGATCCATGTCGCTCGACGAGCTTCCGCAC | | Subcellular location |
|  | R:CTTGCTCACCATGGATCCATCACGCGGGCCGTCGAATGACTGG | |  |
| *OsNADK1pro*:: GUS | F: TGACCATGATTACGAATTCGGGCAAGTTCAAATCAGTGGAAATG | | Expression pattern |
|  | R: ACCCTCAGATCTACCATGGGGCGCGATCCGGCGGTTGGCTCAC | |  |

*OsActin1* (Os03g0718100) and *OsUBQ* (Os02g0634800) were chosen as the internal control.
